# Integrated chemical analysis, metabolic profiling, network pharmacology, molecular docking and toxicity prediction to reveal the active ingredients and their safety of raw and prepared rhubarbs in the treatment of gastric ulcers

**DOI:** 10.3389/fphar.2024.1481091

**Published:** 2024-11-18

**Authors:** Chenxi Wang, Xin Zhao, Jingjing Jiang, Mengqi Jia, Wenqing Shi, Zhenghua Wu, Shiyu Feng, Guorong Fan, Yuefen Lou

**Affiliations:** ^1^ Department of Pharmacy, Shanghai Fourth People’s Hospital Affiliated to Tongji University School of Medicine, Shanghai, China; ^2^ Department of Clinical Pharmacy, Shanghai General Hospital, Shanghai Jiaotong University School of Medicine, Shanghai, China; ^3^ Shanghai University of Finance and Economics, Shanghai, China; ^4^ School of Foreign Studies, Shanghai University of Finance and Economics, Shanghai, China

**Keywords:** raw and prepared rhubarbs, chemical analysis *in vitro*, metabolite profiling *in vivo*, network pharmacology, molecular docking, toxicity prediction

## Abstract

**Background:**

Rhubarb, containing raw rhubarb (RR) and two processed products (steamed rhubarb, SR; carbonized rhubarb, CR), is commonly used in high-doses for the treatment of peptic ulcer, especially gastric ulcer (GU). However, their active ingredients, therapeutic targets, and potential mechanism remain unclear. Meanwhile, the safety of these active ingredients is also worth studying.

**Methods:**

An offline two-dimensional low-pressure liquid chromatography/high-performance liquid chromatography coupled with high resolution mass spectrometry method was applied to identify the chemical constituents of RR, SR, and CR. Then, the plasma and urine samples of rats after oral administration of RR, SR, and CR were studied for metabolite profiling. Based on the analysis of ingredients *in vivo*, the key active constituents, core therapeutic targets and key signaling pathways of RR, SR, and CR against GU were screened *via* network pharmacology and molecular docking. Finally, the efficacy and safety of these key active ingredients were evaluated.

**Results:**

Totally, 183, 120 and 115 compounds were identified or tentatively characterized from RR, SR and CR, respectively. Meanwhile, 190, 182 and 180 components were identified after oral administration of RR, SR and CR. By network pharmacology and molecular docking, torachrysone, hydroxyemodin, 6-methylrhein, rhein and emodin anthrone might be the predominant effective constituents in RR, SR, and CR with AKT1 and EGFR being their key targets during the treatment of GU. Moreover, EGFR/PI3K/AKT signaling pathway might play a crucial role in the therapeutic mechanism of GU. *In silio* ADMET predictions categorized 5 compounds as drugs with good oral bioavailability, but these components may induce liver injury.

**Conclusion:**

Overall, our results not only clarified the active substances and molecular mechanism for enhancing our understanding about the traditional efficacy, but also pay attention to the clinical safety issues of raw and prepared rhubarbs.

## Graphical Abstract

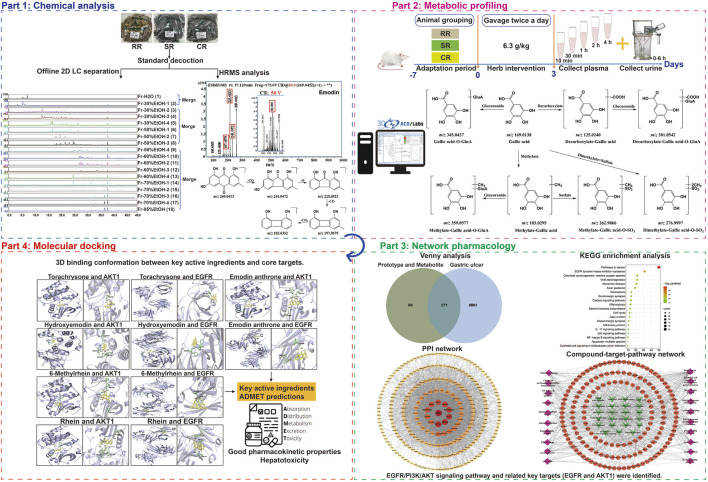

## 1 Introduction

Gastric ulcer (GU) is a common upper gastrointestinal disease worldwide, and its symptoms such as epigastric pain and indigestion seriously affect the quality of life and work efficiency of about 10%–15% of the population in the world (Liu et al., 2022; Bahramikia and Izadi, 2023). GU is characterized by severe damage of gastric mucosa and submucosa, which can cause irritation, inflammation, and increase the risk of gastric cancer (Wang et al., 2022). Generally, it is caused by several factors such as stress, alcohol abuse, over use of nonsteroidal anti-inflammatory drugs (NSAIDs), and *Helicobacter pylori* infection (Shu et al., 2023). At present, GU has been treated with antibiotics, antacids, H2 receptor blockers, and proton-pump inhibitors (Abd-Eldayem et al., 2022). Although these drugs can quickly alleviate clinical symptoms and shorten recovery time, they have some adverse effects, such as nausea, vomiting, stomach pain, headache, and constipation (Liu et al., 2022). Therefore, it is necessary to develop safe, effective, and natural agents. In recent decades, traditional Chinese medicines (TCMs) have unique advantages in treating GU (Abd-Eldayem et al., 2022; Luo J. H. et al., 2023).

Chinese herb rhubarb, the dried roots and rhizomes of Polygonaceae family, comprises Rheum palmatum L., *Rheum tanguticum* Maxim. ex Balf. and Rheum officinale Baill (Zhuang et al., 2020; Zhang et al., 2023). Modern pharmacological research has shown that rhubarb has various pharmacological effects in the gastrointestinal tract, including the protection of intestinal mucosa barrier, the inhibition of *H. pylori*, and the clearance of inflammatory factors (Liu et al., 2016). For a long time, it has been used to treat emergencies such as gastrointestinal bleeding (Zhang et al., 2018; Xiang et al., 2020). Currently, four processed products, namely, raw rhubarb (RR), steamed rhubarb (SR), wine-processed rhubarb (WR), and carbonized rhubarb (CR), are the most commonly used in clinical practice (Zhang J. et al., 2019; Wang et al., 2023). According to the processing theory of “wine processing for uplifting” of TCM, WR is adept at treating upper energizer diseases, such as eye swelling and pain, mouth sores, and ulcers on the tongue. And the stomach belongs to the middle energizer area, so there is literature proving that the astringent and hemostatic effect of WR is the weakest for gastrointestinal bleeding (Wang et al., 2015). Meanwhile, previous researches have demonstrated that RR, SR and CR have therapeutic effects on experimental GUs (Wang, 2023; Li et al., 2019). Therefore, RR, SR and CR were selected as the research objects in this paper. However, the active compounds against GU from the above three rhubarbs remain unclear.

Systematic characterization of the chemical constituents *in vitro* is a critical prerequisite for elucidating the active ingredients of TCMs (Zheng et al., 2021; Wang, 2023). Nowadays, liquid chromatography-high resolution mass spectrometry (LC-HRMS) has become a common method for rapid chemical profiling of TCMs (Shi et al., 2022; Wang H. D. et al., 2024). However, owing to the chemical complexity of TCMs and large differences in content and polarity of each component, the traditional LC-MS method remains a challenge to comprehensively characterize the chemical components in TCMs, especially the minor or even trace existence (Pang et al., 2019; Luo W. J. et al., 2023). To address the problem, multidimensional LC-MS strategy has been proposed (Zhang et al., 2022). For example, offline two-dimensional low-pressure liquid chromatography/high-performance liquid chromatography (2D LPLC/HPLC) coupled with MS approach could enable the isolation and identification of compounds from natural products (Cassien et al., 2021; Zhu et al., 2022). LPLC is a separation technique utilizing different column packings. Middle chromatogram isolated (MCI) GEL CHP20P is one of the most widely used separation materials because of its high capacity and stable quality (Fang et al., 2022; Huang et al., 2023). Therefore, MCI GEL CHP20P column chromatography was chosen for first-dimension separation in our study. We expect that the combination of offline 2D LPLC/HPLC and HRMS in this work can provide strong technical support for elucidating the active compounds of rhubarb.

For complex TCMs, it is well known that only the constituents that enter the circulatory system, rather than all constituents present, are likely to be responsible for the implementation of pharmacological effects (Zhang H. Y. et al., 2019; Li et al., 2022). Collected blood and urine samples are analyzed before and after administration, aiming to discover the exposed substances of TCMs in the body, including prototype components and metabolites (Zhong et al., 2023; Shi et al., 2024). But metabolic analysis *in vivo* does not involve any correlation with pharmacological activity (Li et al., 2022). In recent years, network pharmacology, a “drug-target-disease” interaction analysis approach, has become a hotspot. It can make virtual predictions of pharmacological mechanisms during the treatment with TCMs, so as to make up for the deficiency of metabolite profiling (Xiong et al., 2022; Yang et al., 2022). Hence, an integrated strategy based on metabolic research and network pharmacology was proposed for screening potential active components and targets of TCMs (Zhang et al., 2020).

In the present study, taking RR, SR and CR as examples, we proposed a systematic strategy integrating the characterization of *in vitro* and *in vivo* substances, network pharmacology and molecular docking to reveal anti-GU ingredients, potential key targets and pathways in treating GU. Additionally, the absorption, distribution, metabolism, excretion, and toxicity (ADMET) of these key anti-GU metabolites were further predicted by ADMETlab 2.0 and Deep-PK. It is believed that this work could screen the active compounds as well as their potential targets and pathways, thus laying the foundation for further clarifying the anti-GU mechanism of three processed products of rhubarb. In addition, the safety assessment aimed to provide clinical guidance and minimize the risk of drug-related toxicity for the treatment of GU with rhubarb.

## 2 Materials and methods

### 2.1 Chemicals and reagents

Standard references included emodin, physcion, rhein and chrysophanol, which were purchased from Shanghai Yuanye Biotech. Co., Ltd. (Shanghai, China). RR (batch number: 20230911-1), SR (batch number: 20230814-1) and CR (batch number: 20221019-1) were all purchased from Shanghai Wanshicheng Pharmaceutical Co., Ltd. HPLC grade methanol was acquired from Merck (Darmstadt, Germany). Methanol and acetonitrile of LC-MS grade were provided by Thermo Fisher Scientific Inc. (Waltham, MA, United States). Formic acid (FA) was obtained from ANPEL Laboratory Technologies Inc. (Shanghai, China). Pure water was prepared with a Milli-Q water purification system (Millipore, Bedford, MA, United States). MCI GEL CHP20P high porous polymers (120 μm) was produced by Mitsubishi Chemical Co., Ltd. (Tokyo, Japan). Waters OASIS^®^ HLB solid-phase extraction (SPE) cartridge (1 cc/30 mg) was brought from Waters Technologies Co., Ltd. (Milford, United States).

### 2.2 Sample and standard solution preparation

Rhubarb water decoction was prepared according to the method of standard decoction of medicinal slices. The herbal materials of RR, SR and CR (100 g) were individually soaked in 800 mL water for 30 min. Subsequently, the solution was boiled and then decocted on a small fire for 30 min. Filtering through gauze, the first decoction was thus obtained. Next, the filtered residue was boiled a second time with 700 mL water and then decocted on a small fire for 20 min to obtain the second decoction. Finally, the first and second decoctions were mixed, and concentrated to 200 mg/mL under reduced pressure at 60°C for further analysis. The reference standards were accurately weighed and dissolved in methanol, thus obtain stock solutions with a concentration of 100 μg/mL.

### 2.3 Preliminary segmentation based on offline 2D LPLC/HPLC

A glass column was packed with active MCI GEL CHP20P/P120 (1.5 cm in diameter, 13 cm in height, 1 BV is approximately 25 mL). The column was first rinsed by water until the ethanol was completely cleaned. Then, 6 mL concentrated rhubarb sample was loaded onto the above column equipped with a peristaltic pump (Baoding Lead Fluid Technology Co., Ltd.). The gradient elution was performed with water, 30% ethanol, 50% ethanol, 60% ethanol, 70% ethanol and 95% ethanol (*v/v*), respectively. The flow rate was 3 mL/min, and each gradient elution volume was 4 column volume. The fractions washed with water and 95% ethanol were merged separately, while each other elution process was divided into four sections. The above fractions were concentrated under reduced pressure at 60°C, and then the remaining solution was dried under a flow of nitrogen gas (N_2_). The residue was re-dissolved in an appropriate amount of solvent, vortex for 5 min, and centrifuge at 14,000 rpm at 4°C for 10 min. Finally, the supernatant was injected for HPLC analysis.

The HPLC analysis was acquired on an Ultimate 3000 UHPLC system (Thermo Fisher Scientific, San Jose, CA, United States). The chromatographic separation was carried out on a Hanbon Megres C18 column (250 mm × 4.6 mm, 5 μm) at 30°C with a flow rate of 1 mL/min, and the injection volume was 10 μL. The mobile phase consisted of eluent A (0.1% FA in water, *v/v*) and eluent B (methanol). The optimized gradient elution program was as follows: 0–8 min, 10%–25% B; 8–8.5 min, 25%–30% B; 8.5–21.5 min, 30%–60% B; 21.5–31.5 min, 60%–70% B; 31.5–33.5 min, 70%–95% B; 33.5–38.5 min, 95% B; 38.5–38.6 min, 95%–10% B; 38.6–49 min, 10% B. The UV spectra was recorded at 280 nm.

### 2.4 UPLC-Q-TOF-MS conditions for the chemical profiling in raw and prepared rhubarbs

Qualitative analysis was implemented using an Agilent 1,290 Infinity UPLC system (Milford, MA, United States) coupled with an Agilent 6545 Q-TOF MS, equipped with Dual Agilent Jet Stream electrospray ionization (ESI) source. The LC conditions were the same as described above. The MS system was operated in the negative ESI mode. An Auto MS/MS scan method that included a preferred list (involving all compounds from rhubarb in the literature) was applied to sensitively characterize as many chemical components as possible. The MS parameters were as follows: ion polarity mode, negative; capillary voltage, 3,500 V; sheath gas temperature, 350°C; sheath gas flow, 11 L/min; drying gas flow, 8 L/min; scan range of MS^1^ and MS^2^, 100–1,700 Da; isolation width, 4.0 Da; fixed collision energy, 10/30/60 V; max precursor per cycle, 5; active exclusion, 0.2 min. HRMS data recording was achieved using MassHunter Qualitative Analysis software (version B.08.00, Agilent Technologies).

### 2.5 *In vivo* metabolite profiling

#### 2.5.1 Animals and drug administration

Twenty-four specific pathogen-free (SPF) grade male Sprague-Dawley rats (180 ± 10 g) were purchased from Shanghai SLAC Laboratory Animal Co., Ltd. (Shanghai, China). Rats were adaptively raised in a constant temperature and humid environment for 7 days with free access to standard water and diet. Subsequently, the rats were randomly divided into three groups (*n* = 8): RR group, SR group, and CR group. Five rats in each group were for collecting plasma, while the remaining three were placed in metabolic cages for taking the urine samples. The blank plasma and urine samples were collected before oral administration. The above three groups were administrated RR, SR, and CR twice per day at a dose of 6.3 g/kg (equivalent to five times the clinical dosage) for 3 consecutive days.

#### 2.5.2 Sample collection and pretreatment

Before the last gavage, the rats were fasted but water-free for 12 h. The rats were anesthetized with isoflurane for a short time before blood collection, and the blood samples were collected from the abdominal aorta into heparinized tubes at 0.17, 0.50, 1.0, 2.0, and 4.0 h postdosing. Subsequently, all blood samples were immediately centrifuged (Eppendorf Centrifuge 5424R, Barkhausenweg 1, 22339, Hamburg, Germany) at 7,000 rpm for 10 min at 4°C to obtain plasma, and the supernatants stored at 80°C for subsequent analysis. Additionally, the urine was collected within 6 h after the last administration, and stored at 80°C for later use.

Plasma: 200 μL plasma at six different time points was respectively mixed with 800 μL of cold acetonitrile (containing 0.1% FA) to precipitate protein. After vortexing and centrifuging at 14,000 rpm for 10 min at 4°C, the supernatants were transferred into fresh centrifuge tubes and then dried by N_2_ at room temperature. The residue was reconstituted in 50 μL 70% methanol-water (*v/v*), vortexed for 5 min, and centrifuged at 14,000 rpm at 4°C for 20 min. 10 μL of the supernatant was injected for UPLC-Q-TOF-MS analysis.

Urine: The urine samples were thawed at room temperature and then centrifuged at 4,000 rpm at 4°C for 10 min. Take 1 mL of the supernatant and mix with an equal volume of water for later use. Firstly, the SPE cartridges were activated with 1 mL methanol, followed by 1 mL of water. Then, 1 mL diluted urine sample was loaded onto the pre-activated SPE column and washed with 1 mL water. After drying, the cartridges were eluted by 1 mL of methanol. The methanol eluent was collected, and evaporated to dryness under N_2_. The dried samples were reconstituted with 500 μL of 70% methanol-water (*v/v*), vortexed for 2 min, and then centrifuged at 14,000 rpm for 20 min at 4°C. 10 μL of the supernatant was applied for UPLC-Q-TOF-MS analysis.

#### 2.5.3 UPLC-Q-TOF-MS conditions

The UPLC-Q-TOF-MS conditions were the same as the “*in vitro* chemical analysis” mentioned above.

#### 2.5.4 Data processing strategy

The metabolite identification procedures were as follows: firstly, the raw data of plasma and urine at different times were converted into spectrus format by the Spectra Processor module in the ACD/Labs software. Next, the above data were loaded into the MetaSense processing interface for metabolite identification. Some parameters for metabolite prediction were as follows: species, rat; experiment time, blank, 0.17, 0.50, 1.0, 2.0 and 4.0 h (plasma), blank and 6.0 h (urine); structure/spectrum, mol or. sdf format of the parent structure; “Generate Phase II Metabolites” option was turned on; limit depth of metabolism, 2; probability threshold, 30%; minimum mass, 100. Meanwhile, the following criteria were used to verify and confirm metabolites: 1) remove metabolites that present in the blank experiment; 2) remove metabolites that are only present in one experiment; 3) remove metabolite with slow speed of reaction; 4) remove metabolite with significant fluctuation. To review MetaSense results, the. cfd file was opened by Spectrus DB, and a series of phase I and phase II metabolites were obtained based on the prototype structure. Finally, the obtained metabolites were further verified by MS/MS fragment information.

### 2.6 Network pharmacology analysis

#### 2.6.1 Screening of target genes related to the active components and disease

Firstly, the sdf or mol structures of most compounds were directly searched for in PubChem database (https://pubchem.ncbi.nlm.nih.gov/). While some metabolites need to first find the Pubchem CID on the DCABM-TCM website (http://bionet.ncpsb.org.cn/dcabm-tcm/#/Home), and then obtain their structures by searching for Pubchem CID in the Pubchem database. Subsequently, the comprehensive pharmacokinetics and drug-likeness of absorbed components were evaluated by uploading their structures to the SwissADME platform (http://www.swissadme.ch/), thus the active ingredients of raw and processed rhubarb were obtained. Next, the target prediction of every active component was performed by the Swiss Target Prediction platform (http://www.swisstargetprediction.ch/) and the BATMAN-TCM version 1.0 database (http://bionet.ncpsb.org/batman-tcm/). The target genes associated with gastric ulcers were collected from the GeneCards (https://www.genecards.org/) and OMIM (https://omim.org/) database. The keywords were “gastric ulcer”, “gastrohelcosis”, “gastrohelcoma”, “GU”, and “peptic ulcer”. Lastly, the common targets of active components and disease were screened out using the jvenn plug-in (https://jvenn.toulouse.inra.fr/app/example.html).

#### 2.6.2 Construction of protein-protein interaction (PPI) network

The PPI was achieved based on STRING database (version 12.0, https://cn.string-db.org/), and PPI network was generated according to the ordering of degree values by Cytoscape 3.9.1 software (https://cytoscape.org/).

#### 2.6.3 KEGG pathway and GO enrichment analysis

All targets were subjected to GO function and KEGG pathway enrichment analysis using the Metascape database (https://metascape.org/gp/index.html#/main/step1). GO analysis includes molecular functions (MF), biological processes (BP), and cellular components (CC). Finally, *p*-values < 0.05 was considered statistically significant. The results were visualized by online bioinformatics software (https://www.bioinformatics.com.cn/).

#### 2.6.4 Compound-target-pathway network establishment

A compound-target-pathway network was constructed using the compounds, significantly enriched KEGG pathways, and the relevant targets in each signaling pathway. And use degree as the standard to obtain more important targets and pathways.

### 2.7 Molecular docking validation

First, the sdf format of key active ingredients as ligands was downloaded from PubChem database and saved as pdb format in OpenBabel 3.1.1 software. Meanwhile, the 3D structures of the core targets as protein receptors were obtained from RCSB Protein Data Bank (RCSB PDB, https://www.rcsb.org/). Subsequently, the receptor and ligand PDBs were loaded into the AutoDockTools (Version 1.5.7) and converted to. pdbqt after deleting water and adding hydrogens. These molecules were used for docking analysis to calculate the binding energy between the receptors and ligands. In general, if the binding energy is less than 0 kcal/mol, it indicates that the ligand and the receptor can spontaneously bind. In this study, the binding affinity ≤ − 5.0 kcal/mol was selected as the screening basis. The results were visualized using PyMOL software (version 2.6.0).

### 2.8 Prediction of ADMET properties

Pharmacokinetic parameters related to drug ADMET of key active compounds were predicted by ADMETlab 2.0 (https://admetmesh.scbdd.com/) and Deep-PK online tool (https://biosig.lab.uq.edu.au/deeppk/).

## 3 Results

### 3.1 Preliminary segmentation of raw and prepared rhubarbs based on offline 2D LPLC/HPLC

**FIGURE 1:**
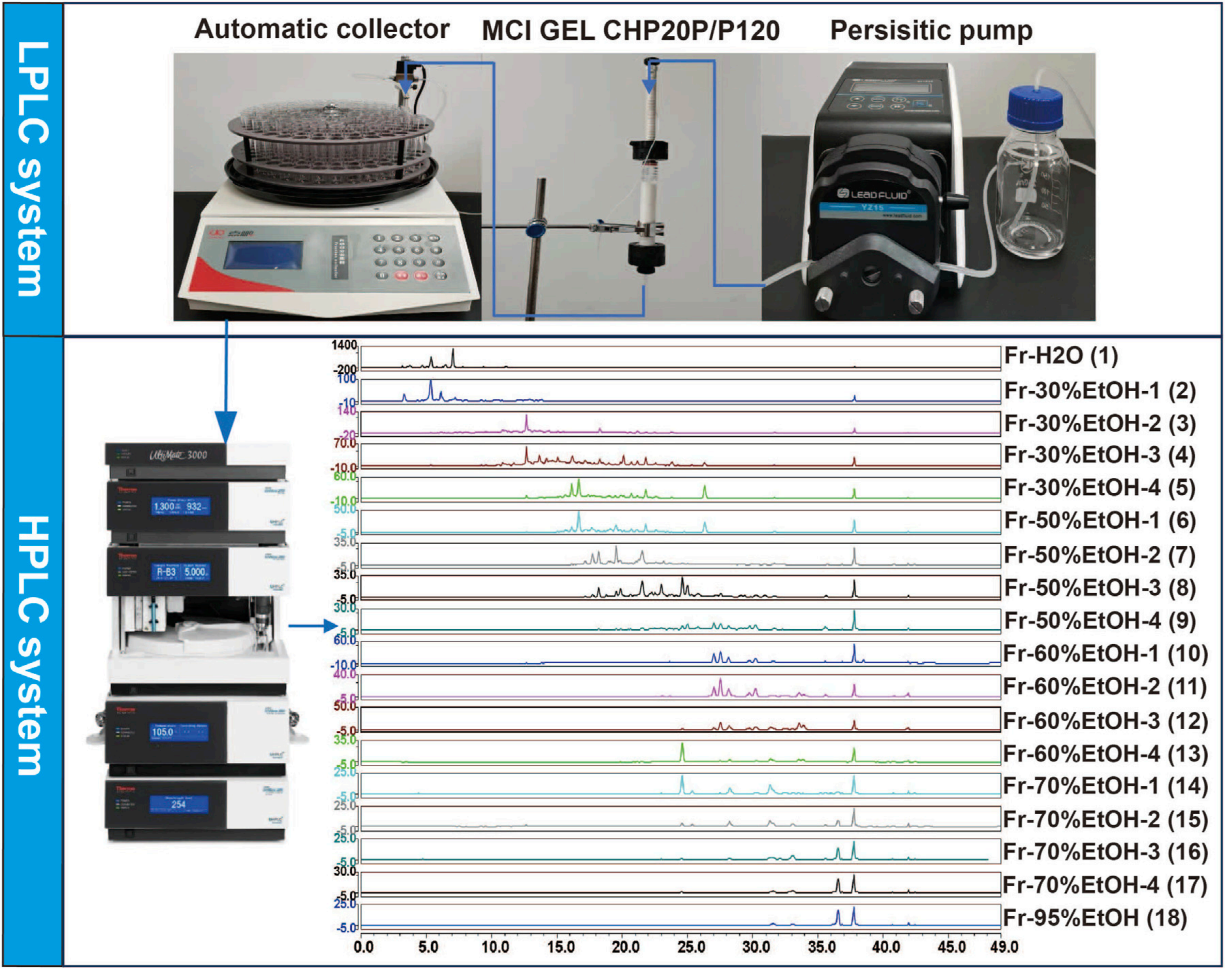
The schematic diagram of the offline 2D LPLC/HPLC system.

**FIGURE 2:**
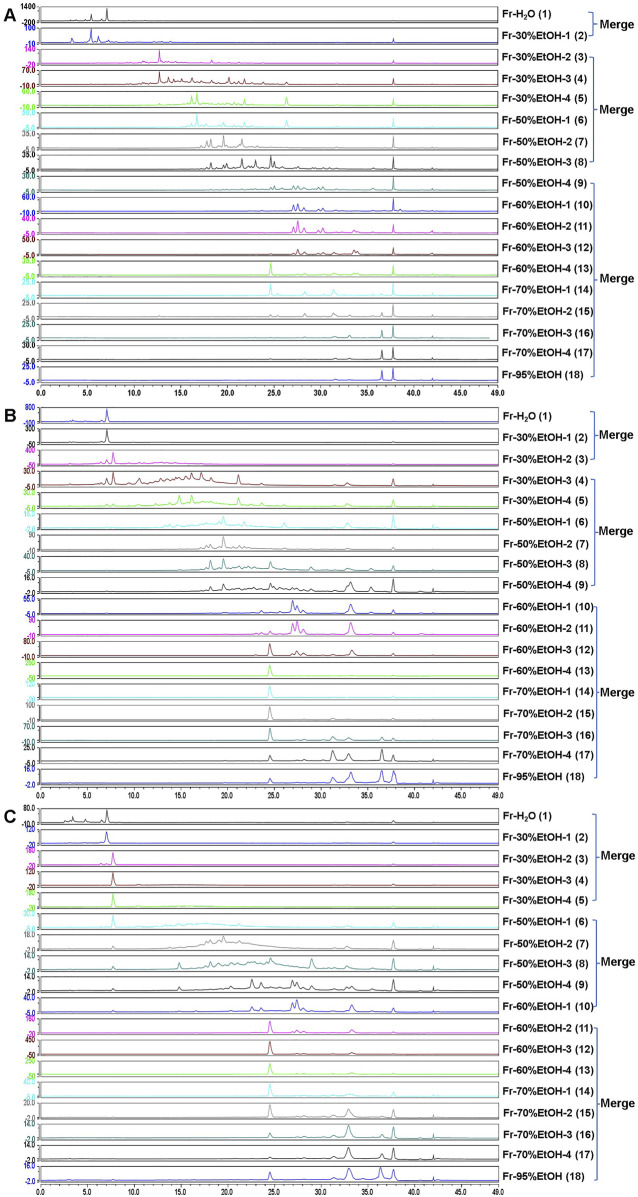
The chromatogram of the RR **(A)** SR **(B)** and CR **(C)** fraction samples.

As shown in Figure 1, an offline 2D LPLC/HPLC system was constructed to visualize the separation process. To achieve the initial separation of the RR, SR and CR, 100 mL elution gradients of 30%, 50%, 60%, 70%, and 95% EtOH, respectively, were applied to elute components. After this separation, eighteen fractions were obtained and further merged into three fractions according to the peak order (Figure 2). Consequently, these fractions were concentrated by N_2_ and subjected to UPLC-Q-TOF-MS analysis.

### 3.2 Systematic characterization of multicomponents in raw and prepared rhubarbs

**FIGURE 3:**
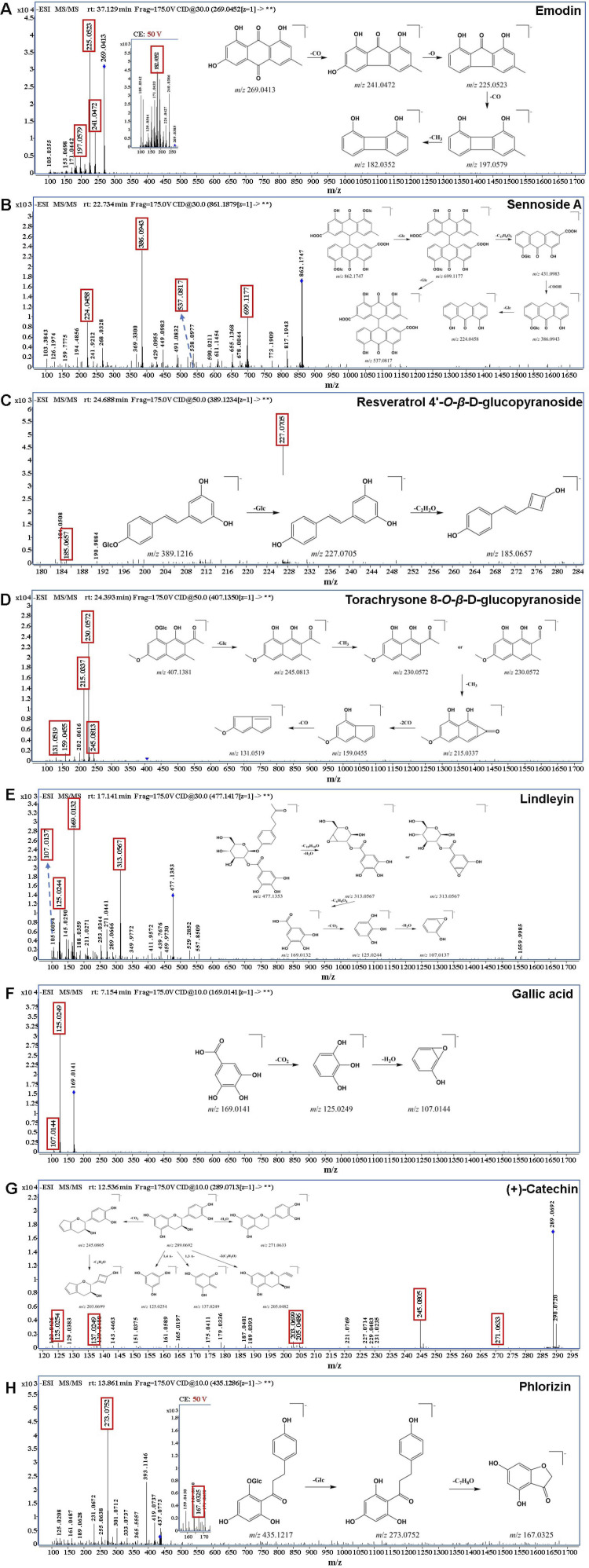
The MS/MS spectrum of typical compounds. **(A)** anthraquinones: emodin; **(B)** anthrones: sennoside A; **(C)** stilbenes: resveratrol 4′-*O*-*β*-D-glucopyranoside; **(D)** naphthalenes: torachrysone 8-*O*-*β*-D-glucopyranoside; **(E)** butyrophenones: isolindleyin; **(F)** tannins: gallic acid; **(G)** tannins: (+)-catechin; **(H)** flavonoids: phlorizin.

All the components in RR, SR and CR were separated by 2D separation to obtain three fractions, which were then respectively analyzed by the UPLC-Q-TOF-MS instrument (Supplementary Figure S1). Raw data were processed using Agilent MassHunter Qualitative analysis software, and the element compositions were predicted with an exact mass number deviation threshold of ±5 ppm. The identification procedure of the chemical components was as follows: firstly, directly and accurately identified by comparing the *t*_*R*_, MS, and MS/MS fragments with reference standards; secondly, identified by comparing the precursor ions, and characteristic fragment ions with literatures and online databases (Metlin, Pubchem and HMDB); thirdly, identified based on characteristic product ions and/or neutral losses that summarized from the analysis of known compounds. As a result, a total of 183, 120 and 115 compounds were detected and tentatively characterized in RR, SR and CR, respectively, involving anthraquinones, anthrones, stilbenes, naphthalenes, butyrophenones, tannins, flavonoids, and others. The detailed compound information was listed in Supplementary Table S1. For anthraquinones, there were emodin, aloe-emodin, physcion, chrysophanol, rhein, and their glycosides. And the anthrones mainly contain sennoside A, sennoside B, sennoside C and sennoside D. For stilbenes, there were resveratrol 4′-*O*-*β*-D-glucopyranoside and rhaponticin. For naphthalenes, there were torachrysone and torachrysone 8-*O*-*β*-D-glucopyranoside. For butyrophenones, there was lindleyin. For tannins, there were gallic acid, (+)-catechin, procyanidin B1 and so on. For flavonoids, there were phlorizin and quercetin. The MS/MS spectrum of typical compounds in each structural type were shown in Figure 3. On the other hand, in this work, compared with the traditional one-dimensional chromatographic system, 38 chemical compounds were characterized by combining offline 2D LPLC/HPLC system with HRMS (Supplementary Table S1). The result indicated that the additional 2D chromatographic separation could expand the peak capacity.

### 3.3 Identification of the prototypes and metabolites in rat plasma and urine of raw and prepared rhubarbs

**FIGURE 4:**
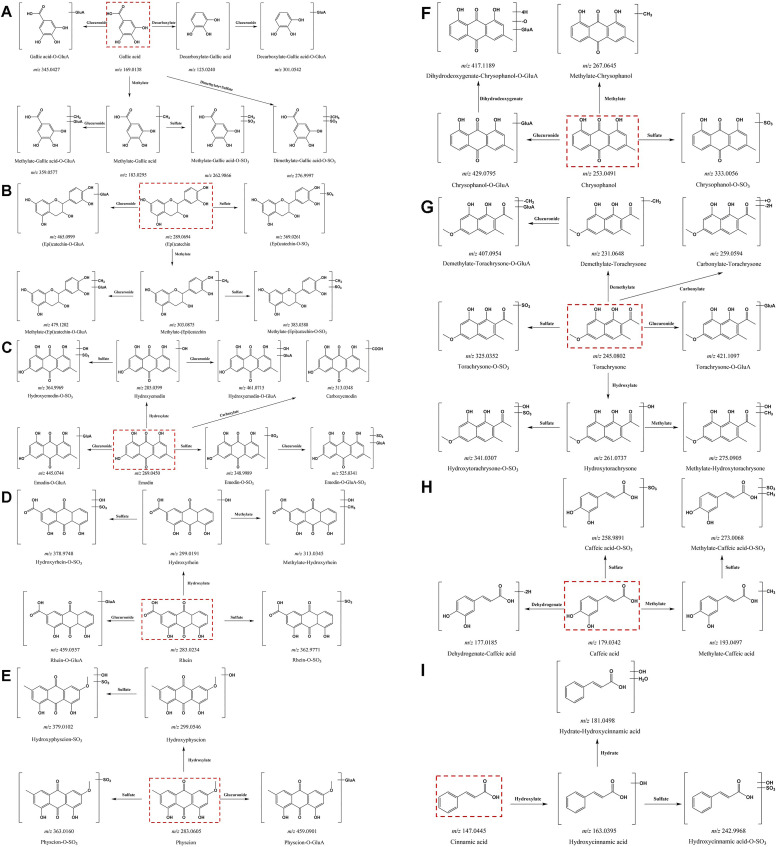
Metabolic pathways of all prototype components in rats. **(A)** gallic acid; **(B)** (epi) catechin; **(C)** emodin; **(D)** rhein; **(E)** physcion; **(F)** chrysophanol; **(G)** torachrysone; **(H)** caffeic acid; **(I)** cinnamic acid.

To evaluate the metabolism of absorbable constituents in RR, SR and CR *in vivo*, the prototypes and metabolites found in the dosed plasma and urine were analyzed using UPLC-Q-TOF-MS. Firstly, the blank plasma (urine) and Chinese medicinal materials were used as negative and positive controls, respectively. The extracted ion chromatographic peaks, which simultaneously appeared for the dosed plasma (urine) and TCMs but not the blank plasma, were identified as the absorbed prototype ingredients. In addition, compounds that could be detected in the dosed plasma (urine) but not in the blank plasma and TCMs, and had the same characteristic ion fragments with prototype components, were considered as metabolites. According to the above criterion, 70, 67 and 66 prototypes were identified as the absorbed ingredients in the dosed plasma and urine of RR, SR and CR. Meanwhile, 120, 115 and 114 metabolites derived from the prototype constituents in rat plasma and urine of RR, SR and CR were inferred by the ACD/Labs software. It mainly involved phase I metabolism such as hydroxylation, hydrogenation, hydration, desaturation, demethylation, dehydrogenation, deoxygenation, carboxylation and phase II metabolism such as methylation, sulfation, glucuronidation. All identified prototypes and metabolites were displayed in Supplementary Table S2. The table showed the name, *t*_*R*_, measured *m/z*, ppm, formula and fragments. And the metabolic pathway of all compounds (gallic acid, (epi) catechin, emodin, rhein, physcion, chrysophanol, torachrysone, caffeic acid and cinnamic acid) was constructed (Figure 4). Ultimately, we identified 143 metabolites of RR in the plasma and 159 metabolites of RR in the urine. Furthermore, in SR, a total of 131 metabolites were identified in the plasma and 140 metabolites were identified in the urine. In addition, 133 metabolites of CR were identified in the plasma and 123 metabolites of CR were identified in the urine.

**FIGURE 5:**
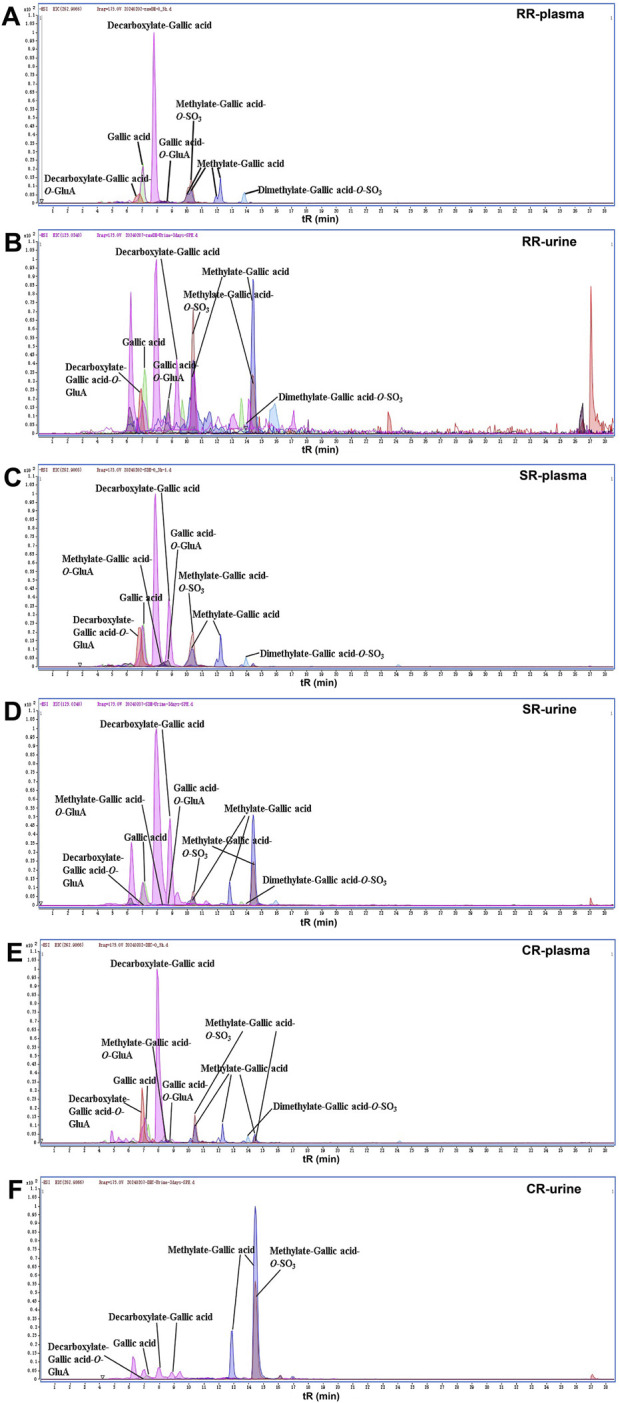
The EICs of gallic acid and its metabolites in rats. **(A)** RR-plasma; **(B)** RR-urine; **(C)** SR-plasma; **(D)** SR-urine; **(E)** CR-plasma; **(F)** CR-urine.

Here, we described the corresponding metabolic pathways in rats using gallic acid as an example (Figure 4A). The extracted ion chromatograms (EICs) of both prototypes and metabolites after oral administration of RR, SR and CR by UPLC-Q-TOF-MS in negative ion mode were displayed in Figure 5, respectively. Gallic acid presented high-intensity [M-H]^-^ ion at *m/z* 169.0138. Its fragment ion at *m/z* 125.0247 was produced by the loss of a CO_2_ (44 Da). Thus, the [M-H]^-^ ion at *m/z* 125.0244 was characterized as decarboxylate-gallic acid. Furthermore, the precursor ion at *m/z* 301.0542 was 176 Da (GluA) greater than the molecular ion of *m/z* 125.0244, indicating that it was a glucuronidation product of decarboxylate-gallic acid (decarboxylate-gallic acid-*O*-GluA). Also, the [M-H]^-^ ion at *m/z* 345.0427 is 176 Da (GluA) more than the prototype gallic acid, and it contained the characteristic fragment ions at *m/z* 169.0132 and *m/z* 125.0248. Hence, it was defined as gallic acid-*O*-GluA. The ion at *m/z* 183.0295 was 14 Da larger than gallic acid, suggesting that it was a metabolite from methylation of gallic acid. Additionally, the molecular ions at *m/z* 359.0577 and *m/z* 262.9866 were respectively deduced to be the glucuronidated and sulfated product of methylate-gallic acid because their molecular weights were respectively 176 Da (GluA) and 80 Da (SO_3_) higher than that of methylate-gallic acid. Besides, the [M-H]^-^ ion at *m/z* 276.9997 was two CH_2_ (28 Da) and one SO_3_ (80 Da) larger than *m/z* 169.0138 gallic acid. Its MS/MS spectrum displayed a few unique product ions at *m/z* 197.0440 (-SO_3_), 183.0261 (-SO_3_-CH_2_), 168.0058 (-SO_3_- 2CH_2_), and 124.0162 (-SO_3_-2CH_2_-CO_2_). Therefore, it was assigned as dimethylate-gallic acid-*O*-SO_3_.

### 3.4 Screening of potential active components, core targets and key pathways

**FIGURE 6:**
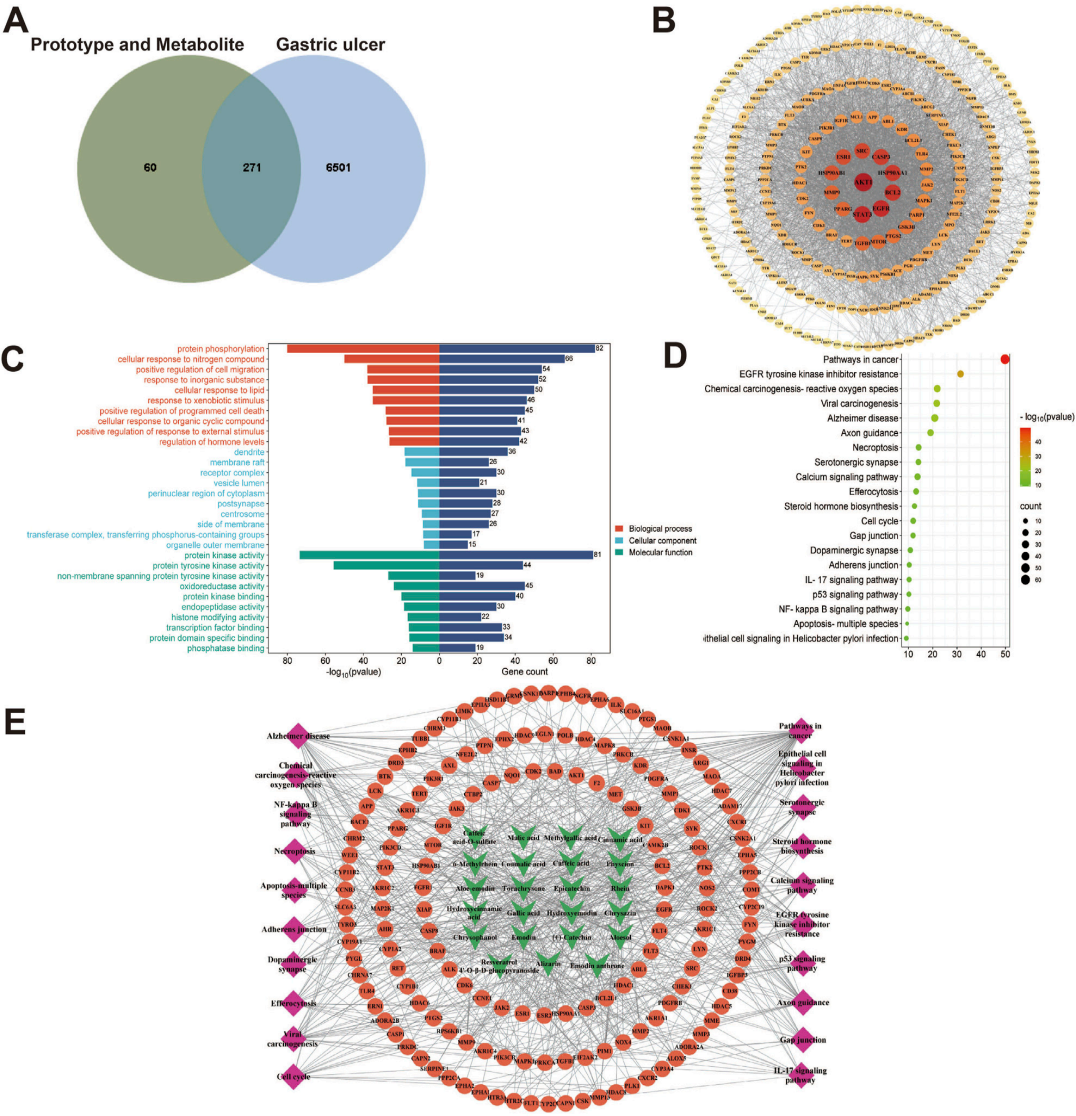
Network analysis of three processed rhubarb products against GU. **(A)** Venn diagram of 271 intersection targets of rhubarb and GU; **(B)** PPI network exhibited the hub genes; **(C)** GO enrichment analysis; **(D)** KEGG pathway enrichment analysis; **(E)** Compound-target-pathway network. Green nodes refer to the active compounds, orange nodes refer to the targets, pink nodes refer to the top 20 KEGG pathway.

In our present analysis, 23 potential active ingredients were recognized in RR, SR and CR. All active components met Lipinski’s rule of five with no violations and possessed excellent gastrointestinal absorption. Predicted bioavailability scores were all greater than 0.5, supporting the above active compounds as good orally bioavailable drugs. The more information on active compounds of RR, SR and CR screened by SwissADME was listed in Supplementary Table S3. A total of 331 targets were obtained from the Swiss Target Prediction and BATMAN-TCM database. Meanwhile, 6,772 targets related to GU were collected from the Genecards and OMIM database. Finally, 271 intersection targets were summarized to be possible anti-GU targets of the RR, SR and CR (Figure 6A). A PPI network of the above common targets was constructed and the network consisted of 271 nodes and 3,716 edges totally (Figure 6B). Among the 271 overlapped targets, the top 5 targets (AKT1, STAT3, EGFR, BCL2, and HSP90AA1) were selected as the key targets based on the values of degree (Table 1).

**TABLE 1:** The top 5 targets obtained from PPI nerwork.

Targets	Degree
AKT1	152
STAT3	130
EGFR	127
BCL2	122
HSP90AA1	119

Subsequently, the 271 overlapping targets were used to perform GO and KEGG pathway enrichment analysis. Based on the GO biological processes analysis, protein phosphorylation, cellular response to nitrogen compound, positive regulation of cell migration and others were significantly enriched (Figure 6C). Moreover, the top 10 terms in GO molecular functions analysis were protein kinase activity, protein tyrosine kinase activity, non-membrane spanning protein tyrosine kinase activity, etc (Figure 6C). Besides, the result of GO cellular components enrichment showed that it was mainly related to dendrite, membrane raft, receptor complex, vesicle lumen and etc (Figure 6C). Furthermore, the top important 20 items of the KEGG result were selected to draw the bubble chart (Figure 6D). According to the KEGG enrichment analysis, the signaling pathways mainly involved the pathways in cancer, EGFR tyrosine kinase inhibitor resistance, chemical carcinogenesis-reactive oxygen species and so on.

**FIGURE 7:**
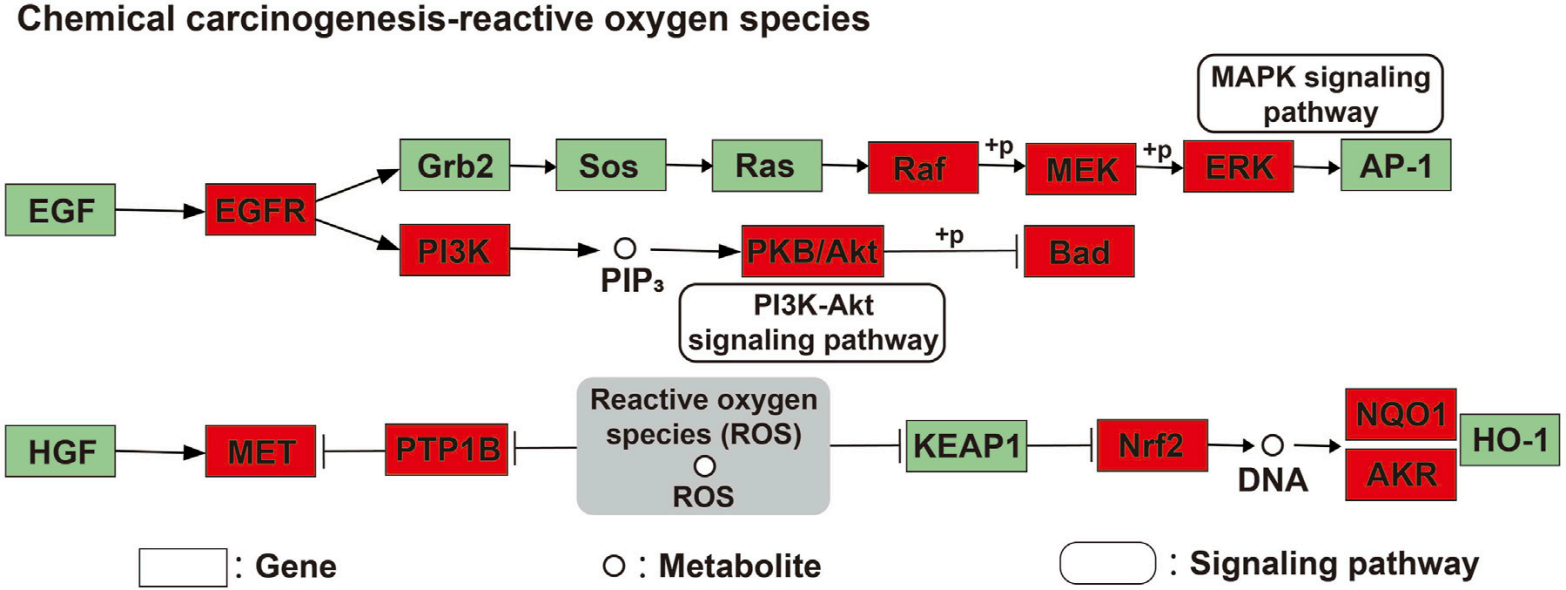
The integration and visualization of targets in chemical carcinogenesis-reactive oxygen species pathway. Red represented the gathered potential targets.

Finally, a compound-target-pathway network was constructed to clarify the mechanism of three processed rhubarb products against GU (Figure 6E). And took the degree as the criterion to get more important components, targets and pathway. Torachrysone, hydroxyemodin, physcion, chrysophanol, 6-methylrhein, caffeic acid, rhein, emodin, emodin anthrone and cinnamic acid may be the key active components of three processed rhubarb products that could improve GU (Table 2). In the network, the top 5 gene targets based on the values of degree included EGFR, BCL2, ESR2, SRC and MET (Table 3). Additionally, the ranking result of the degree value of KEGG pathway showed that chemical carcinogenesis-reactive oxygen species pathway may be more important pathway (Table 4). Then, the KEGG mapping (https://www.kegg.jp/kegg/kegg1b.html) was used to map the chemical carcinogenesis reactive oxygen species pathway. As shown in Figure 7, the potential targets (labeled red) were gathered in the chemical carcinogenesis-reactive oxygen species. It was found that EGFR/PI3K/AKT, RAF/MEK/ERK/MAPK and ROS/NRF2/NQO1/AKR signaling pathways might be implicated in this therapeutic mechanism. Among them, EGFR/PI3K/AKT signaling pathway was the most significant pathway and the relevant genes EGFR and AKT1 were the key targets.

**TABLE 2:** The top 10 compounds obtained from compound-target-pathway network.

Compounds	Degree
Torachrysone	57
Hydroxyemodin	52
Physcion	41
Chrysophanol	40
6-Methylrhein	32
Caffeic acid	27
Rhein	25
Emodin	23
Emodin anthrone	21
Cinnamic acid	15

**TABLE 3:** The top 5 targets obtained from compound-target-pathway network.

Targets	Degree
EGFR	17
BCL2	15
ESR2	14
SRC	12
MET	12

**TABLE 4:** The top 10 pathways obtained from compound-target-pathway network.

Pathways	Degree
Pathways in cancer	62
Alzheimer disease	32
Chemical carcinogenesis-reactive oxygen species	27
Viral carcinogenesis	26
EGFR tyrosine kinase inhibitor resistance	25
Axon guidance	23
Calcium signaling pathway	21
Necroptosis	18
Efferocytosis	17
Serotonergic synapse	16

### 3.5 Molecular docking evaluation

**FIGURE 8:**
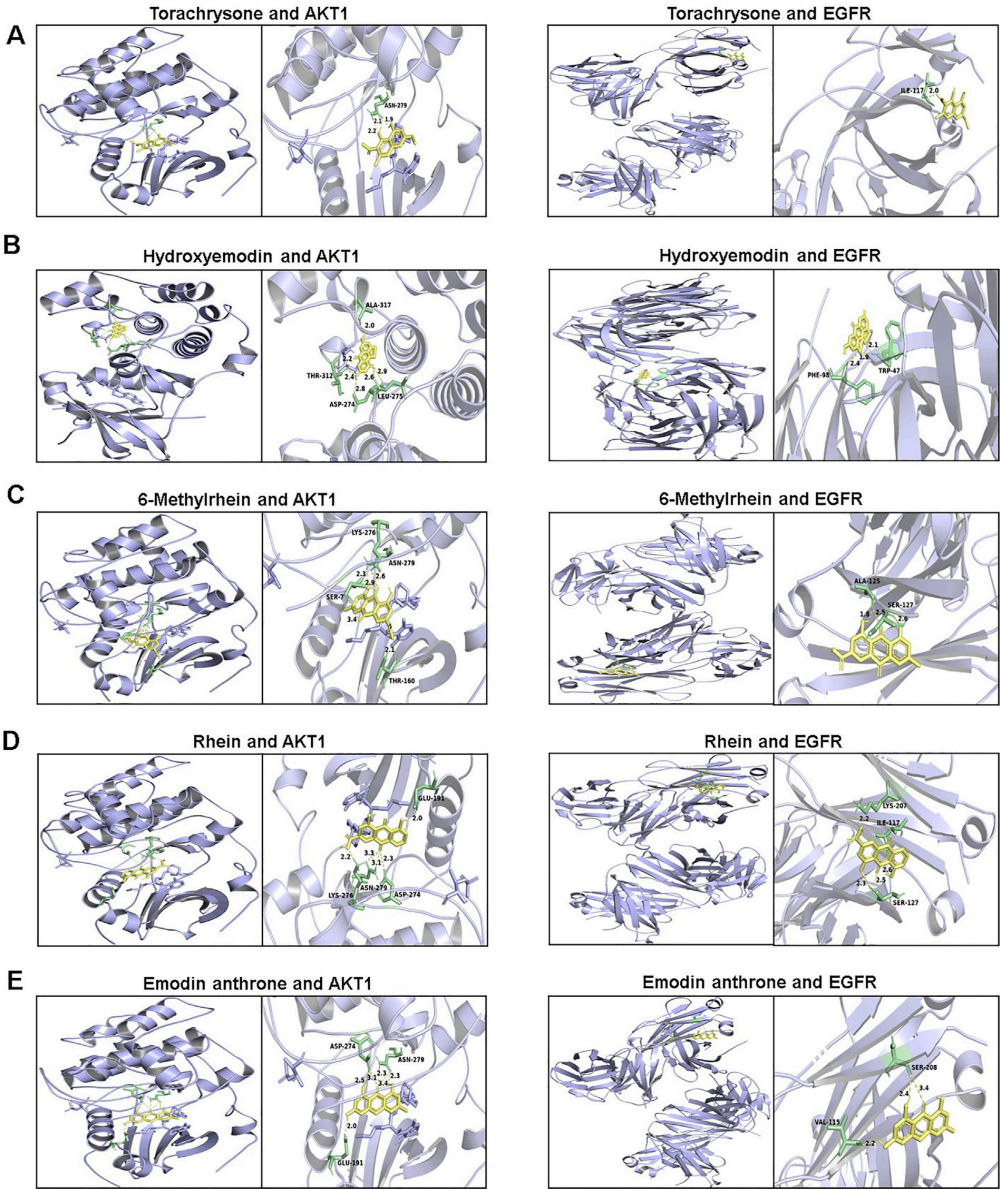
The 3D interaction diagrams between active components and key targets. **(A)** torachrysone; **(B)** hydroxyemodin; **(C)** 6-methylrhein; **(D)** rhein; **(E)** emodin anthrone.

To further explore the possibility of interaction between active ingredients and the core targets, the top 10 compounds in the compound-target-pathway network were selected to perform molecular docking with AKT1 and EGFR, respectively. The docking results indicated that the target had certain binding affinity with the compound, in which torachrysone, hydroxyemodin, 6-methylrhein, rhein and emodin anthrone had a higher binding energy with each target (Table 5). Moreover, the receptor-ligand binding site and hydrogen bond interaction between the above key active compounds and core targets were analyzed and visualized using PyMOL (Figure 8). In the interaction with AKT1, torachrysone formed hydrogen bonds with ASN-279 amino acid residue, hydroxyemodin formed hydrogen bonds with ALA-317, THR-312, ASP-274, and LEU-275, 6-methylrhein formed hydrogen bonds with LYS-276, ASN-279, SER-7, and THR-160, rhein formed hydrogen bonds with GLU-191, ASN-279, LYS-276, and ASP-274, and emodin anthrone formed hydrogen bonds with ASP-274, ASN-279, and GLU-191. In the interaction with EGFR, torachrysone made hydrogen-bonding interaction with ILE-117, hydroxyemodin made hydrogen-bonding interaction with PHE-98 and TRP-47, 6-methylrhein made hydrogen-bonding interaction with ALA-125 and SER-127, rhein made hydrogen-bonding interaction with LYS-207, ILE-117, and SER-127, and emodin anthrone made hydrogen-bonding interaction with SER-208 and VAL-115.

**TABLE 5:** Molecular docking results of 10 active compounds with target proteins.

Ligand	Binding energy (kcal/mol)	
	AKT1 (PDB ID: 3QKL)	EGFR (PDB ID: 3P0V)
Torachrysone	−6.57	−5.01
Hydroxyemodin	−12.39	−8.48
Physcion	−7.89	−4.74
Chrysophanol	−7.66	−4.80
6-Methylrhein	−7.39	−5.75
Caffeic acid	−5.07	−3.79
Rhein	−7.85	−5.50
Emodin	−8.18	−4.76
Emodin anthrone	−8.01	−5.17
Cinnamic acid	−6.12	−4.91

### 3.6 ADMET properties of 5 key active compounds

To evaluate the druggability and safety of 5 key active ingredients, the ADMET properties were assessed. The assessment results were shown in Table 6. In the aspect of absorption, no compounds were defined as Pgp-substrates. In terms of distribution, no compounds could permeabilize the BBB (Blood-Brain Barrier). With regard to metabolism, torachrysone and emodin anthrone were both the inhibitors of CYP1A2, CYP2C19, CYP2C9, CYP2D6 and CYP3A4 enzymes. Hydroxyemodin may work as the inhibitor of CYP1A2, CYP2C19 and CYP2D6 enzymes. However, 6-methylrhein and rhein had been found to be non-inhibitors of any enzyme. Similarly, all 5 key active compounds were the substrates of CYP2C9 enzyme and only torachrysone was the substrate of CYP1A2 enzyme. The predicted total clearances (CL) of torachrysone, hydroxyemodin and emodin anthrone were moderate, while 6-methylrhein and rhein were at a low level. The toxicity prediction results demonstrated that 5 key active ingredients all posed a risk of drug-induced liver injury.

**TABLE 6:** Predicted ADMET properties of 5 key active compounds.

Compounds	Absorption	Distribution	Metabolism										Excretion	Toxicity	
	Pgp-substrate	BBB Penetration	CYP1A2		CYP2C19		CYP2C9		CYP2D6		CYP3A4		CL	Rat oral acute toxicity	Liver injury
			Inhibitor	Substrate	Inhibitor	Substrate	Inhibitor	Substrate	Inhibitor	Substrate	Inhibitor	Substrate			
Torachrysone	No	No	Yes	Yes	Yes	No	Yes	Yes	Yes	No	Yes	No	Moderate	No	Yes
Hydroxyemodin	No	No	Yes	No	Yes	No	No	Yes	Yes	No	No	No	Moderate	No	Yes
6-Methylrhein	No	No	No	No	No	No	No	Yes	No	No	No	No	Low	No	Yes
Rhein	No	No	No	No	No	No	No	Yes	No	No	No	No	Low	No	Yes
Emodin anthrone	No	No	Yes	No	Yes	No	Yes	Yes	Yes	No	Yes	No	Moderate	No	Yes

## 4 Discussion

GU is the most common disorder of the digestive system (Dinat et al., 2023). Numerous studies indicate that TCMs are new sources of drugs with promising results in treating GU (Bi et al., 2014; Bahramikia and Izadi, 2023). Rhubarb has been extensively used for the treatment of gastrointestinal diseases during clinical practice, such as gastrointestinal dysfunction, GU bleeding, and stress induced-GU (Zhang et al., 2018; Xiang et al., 2020). Additionally, previous research has suggested that raw and prepared rhubarbs have protective effect on various experimental GU (Wang, 2023; Li et al., 2019). And exploring the active constituents is the basis for elucidating the pharmacological mechanism of TCMs. However, the complex chemical composition of TCMs is a major challenge for researchers (Tang et al., 2022). Hence, it is necessary to utilize modern science and technology to determine the active ingredients in the TCMs. Currently, more and more strategies including phytochemistry, serum pharmacochemistry, network pharmacology, multi-omics and association analysis were put forward (Hu et al., 2023; Wang Y. J. et al., 2024). Nonetheless, each technique still has its own advantages and limitations (Lu et al., 2022). Therefore, a comprehensive approach should be proposed to investigate the active components of the TCMs. In this study, we combined phytochemical composition *in vitro*, metabolite profiling *in vivo*, network pharmacology, and molecular docking to elucidate the bioactive compounds, potential targets related to GU and their signaling pathways in RR, SR and CR.

To identify the compositions of RR, SR and CR, an offline 2D LPLC/HPLC combined with MS system was employed for compound separation and identification. By this technique, a total of 184, 121 and 116 compounds were identified or tentatively characterized from RR, SR and CR, respectively. As we know, only those components that can migrate into blood have the opportunity to exert the therapeutic effects. In general, the absorbed components and/or metabolites are regarded as potential pharmacodynamic substances (Zhang et al., 2019; Dai and Sun, 2022; Li et al., 2022). Therefore, the systematic metabolism study on rhubarb *in vivo* is indispensable. In this study, 196 (72 prototypes as well as 124 metabolites), 189 (68 prototypes as well as 121 metabolites) and 188 (67 prototypes as well as 121 metabolites) components were identified in RR, SR, and CR, respectively. Although the quantities were different, the phase I and II metabolic transformation types in RR, SR, and CR were the same. Among them, the absorbed prototype constituents mainly included gallic acid, (epi) catechin, emodin, rhein, physcion, chrysophanol, torachrysone, caffeic acid, cinnamic acid, and their isomers. Also, based on HRMS data and related literature (Xiang et al., 2016), the major metabolic pathways of gallic acid were glucuronidation, sulfation, decarboxylation, and methylation. For the (epi) catechin-related metabolites, the biotransformation pathways included glucuronidation, sulfation, and methylation (Xiang et al., 2016). Emodin could be converted into glucuronided, sulfated, decarboxylated, and hydroxylated metabolites (Yi et al., 2018; Zhang et al., 2019). The predominant metabolic pathways of rhein were glucuronidation, sulfation, methylation, and hydroxylation (Yi et al., 2018). The physcion-ralated metabolic reactions mainly involved glucuronidation, sulfation, and hydroxylation (Xu et al., 2018). The main metabolic reactions of chrysophanol were glucuronidation, sulfation, hydrogenation, deoxygenation, and methylation (Xu et al., 2018). For the torachrysone-related metabolites, the biotransformation pathways included glucuronidation, sulfation, hydroxylation, methylation, and demethylation (Liu et al., 2014). Caffeic acid could be converted into sulfated, methylated, and dehydrogenated metabolites (Yi et al., 2018). Furthermore, sulfation, hydroxylation, and hydration were found to occur in the metabolism process of cinnamic acid (El-Hawary et al., 2021).

Network pharmacology is an efficient tool for finding active substances from numerous components contained in TCMs based on a holistic perspective (Shen et al., 2023; Duan et al., 2024). In recent years, there is an increasing amount of research to unveil active material basis and molecular mechanisms of TCMs for their traditional functions via network pharmacology in combination with metabolite profiling. The combination of these two approaches enables break through the limitations of TCMs, more accurately reflect the true effects of TCMs in the body, and provide a new perspective and idea for the research of TCMs (Deng et al., 2024; Zhao et al., 2024). Based on this integrated analysis, taking the above substances in rat plasma and urine as the research object, further network pharmacology and molecular docking showed that a total of 5 chemical compounds closely related to multiple key targets, such as torachrysone, hydroxyemodin, 6-methylrhein, rhein and emodin anthrone, were considered as material basis of three processed rhubarb products for the treatment of GU. Subsequently, *in silio* ADMET predictions of the above 5 compounds indicated that they showed good oral bioavailability, but may have side effect of hepatotoxicity. Although the “*You Gu Wu Yun*” (with reason but without death) theory was still followed in clinical practice, the safety of rhubarb still led to public concern (Fang et al., 2011). Thus, this study suggested that it is important to focus on monitoring the function of non-pathological tissues (such as the liver). Moreover, interestingly, we found that the peak areas of torachrysone and emodin anthrone in RR were much higher than those in SR and CR (Supplementary Figure S2). Pharmacokinetic results also demonstrated that the content of rhein was higher in RR than in SR (Zhang et al., 2019; Zhou et al., 2022). The above results were basically consistent with the TCM theory of toxicity-attenuating effect of processing.

As predicted by our compound-target-pathway network, the EGFR/PI3K/AKT signaling pathway was determined to be the most relevant pathway to the effects of three processed rhubarb products on GU. Numerous studies have suggested that EGFR (epidermal growth factor receptor) is closely associated with gastrointestinal diseases. It not only participates in cell proliferation but also in several important processes, including apoptosis, invasion, angiogenesis, and metastasis (Lee et al., 2009). In humans, EGFR is overexpressed in gastric cancer compared with normal gastric tissue (George et al., 2020). Meanwhile, the PI3K/AKT signaling also plays a critical role in cell proliferation, migration and survival as well as a key factor in GU healing (Zhou et al., 2023). Previous work has shown that PI3K/AKT signaling pathway could regulate cell apoptosis and inflammation to achieve the treatment of GU (Liu et al., 2022). Interestingly, the PI3K/AKT pathway is regulated by a variety of growth factors (i.e., EGF). EGF bind to its receptor and trigger cell proliferation, migration, and survival by activating the PI3K/AKT pathway (Tarnawski and Ahluwala, 2021). Therefore, one of the mechanisms of the anti-GU activity of rhubarb may involve its effect on EGFR/PI3K/AKT signaling pathway. Most notably, AKT1 and EGFR participate in the signaling pathway. Based on this, the EGFR/PI3K/AKT pathway-related targets were selected for further validation by molecular docking. Our results revealed a strong affinity between 5 key active compounds and 2 core targets.

To the best of our knowledge, this represents the first assessment and investigation of the efficacy and safety of raw and two prepared rhubarbs in the treatment of GU systematically and comprehensively. However, some limitations should be mentioned in this study. The reliability and accuracy of our active ingredients, core target genes, key signaling pathway and their toxicity depend to some extent on the quality of the database data. It is imperative to validate these findings through *in vivo* and *in vitro* verified experiments.

## 5 Conclusion

This research evaluated the efficacy and safety of raw and two prepared rhubarbs in the treatment of GU. On the one hand, a systematic strategy integrating the comprehensive characterization of *in vitro* and *in vivo* components, network pharmacology and molecular docking was proposed to screen the active ingredients, explore the key targets and predict potential mechanisms of RR, SR and CR in treating GU. On the other hand, the druggability and safety of these key active compounds were assessed. We discovered that the main active compounds contained in RR, SR and CR included torachrysone, hydroxyemodin, 6-methylrhein, rhein and emodin anthrone. Meanwhile, the integrated analysis revealed 2 key targets (AKT1 and EGFR) as well as related EGFR/PI3K/AKT signaling pathway. In the druggability and safety evaluation, the above 5 compounds had good pharmacokinetic properties, but may possess the risk of hepatotoxicity. Our results provided data and theoretical basis for an in-depth study on the anti-GU mechanism of the RR, SR and CR. Furthermore, this study could provide a hint that clinicians may need to balance the benefits and risks of raw and two prepared rhubarbs treatment for GU.

## Data Availability

The original contributions presented in the study are included in the article/Supplementary Material, further inquiries can be directed to the corresponding authors.
