# Supplementary material for: Integrated chemical analysis, metabolic profiling, network pharmacology, molecular docking and toxicity prediction to reveal the active ingredients and their safety of raw and prepared rhubarbs in the treatment of gastric ulcers
Source: Front Pharmacol. 2024 Nov 18;15:1481091. doi: 10.3389/fphar.2024.1481091 (PMC11608977; doi:10.3389/fphar.2024.1481091)
Supplement: Supplementary file 1 [file DataSheet1.docx]

Supplementary Material

# Supplementary Figures and Tables

## Supplementary Figures


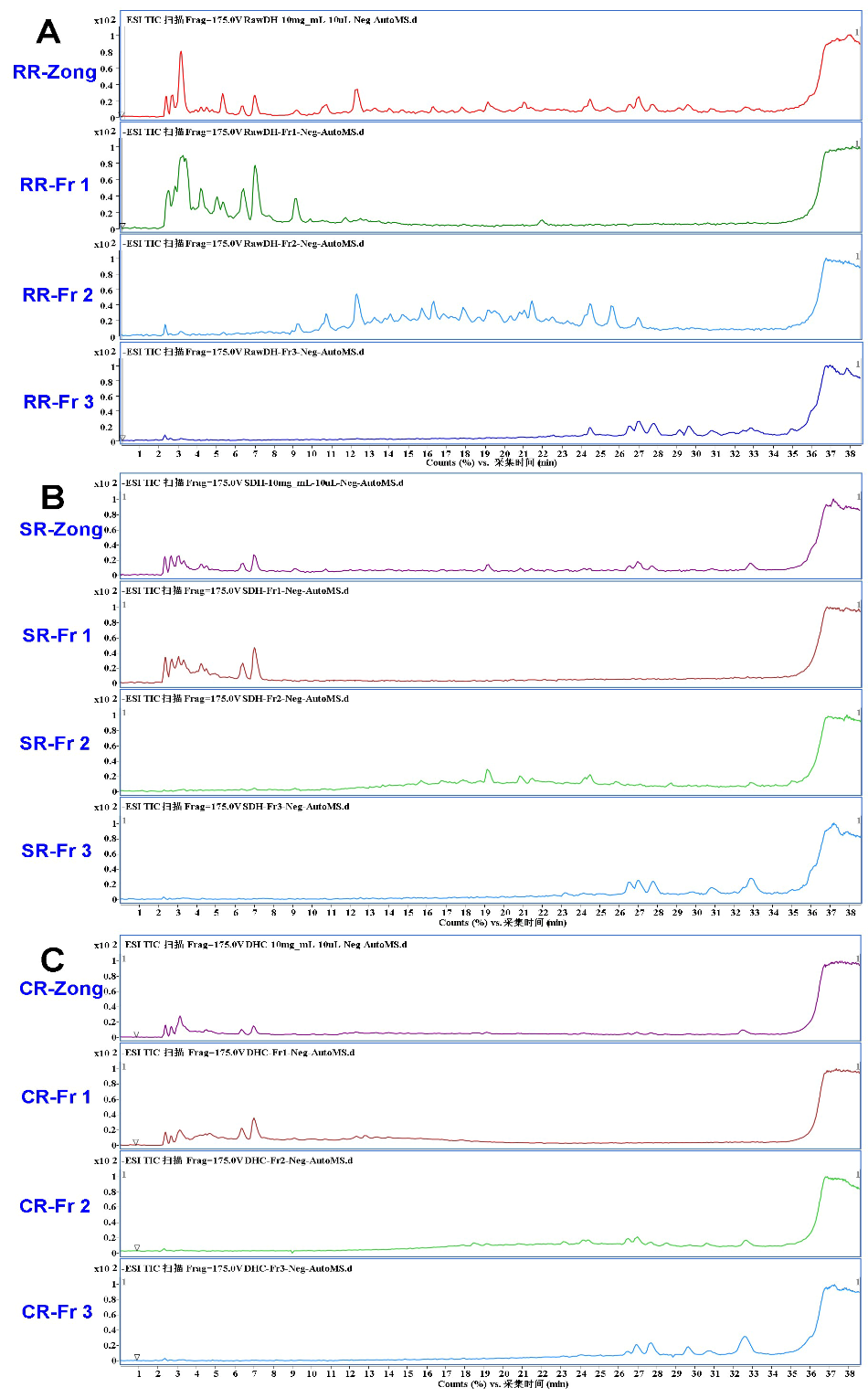


**Supplementary Figure 1.** The total ion chromatograms (TICs) of RR (A), SR (B) and CR (C) samples and their three fractions determined by UPLC-Q-TOF-MS in negative ion mode.


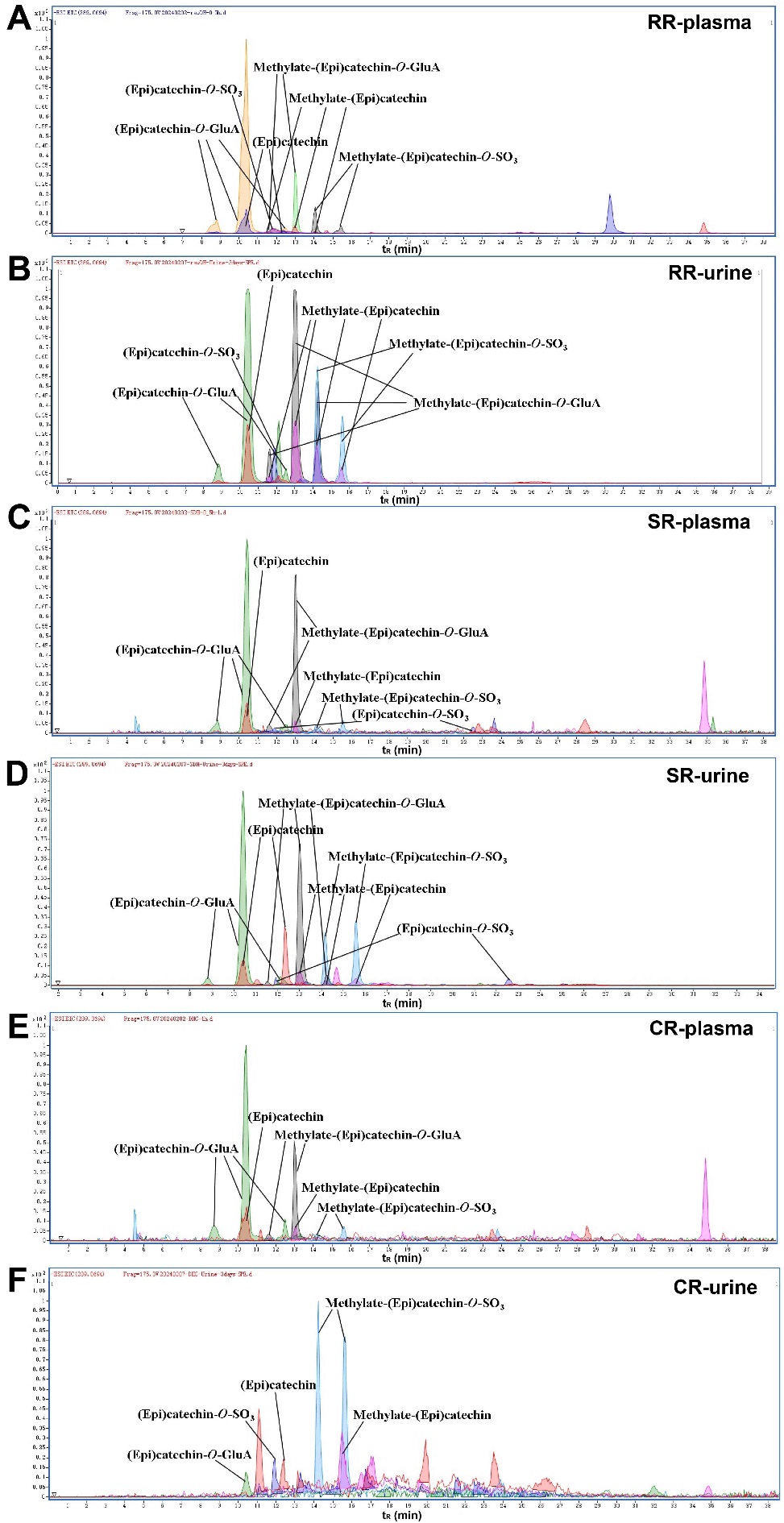


**Supplementary Figure 2.** The EICs of (epi)catechin and its metabolites in rats.


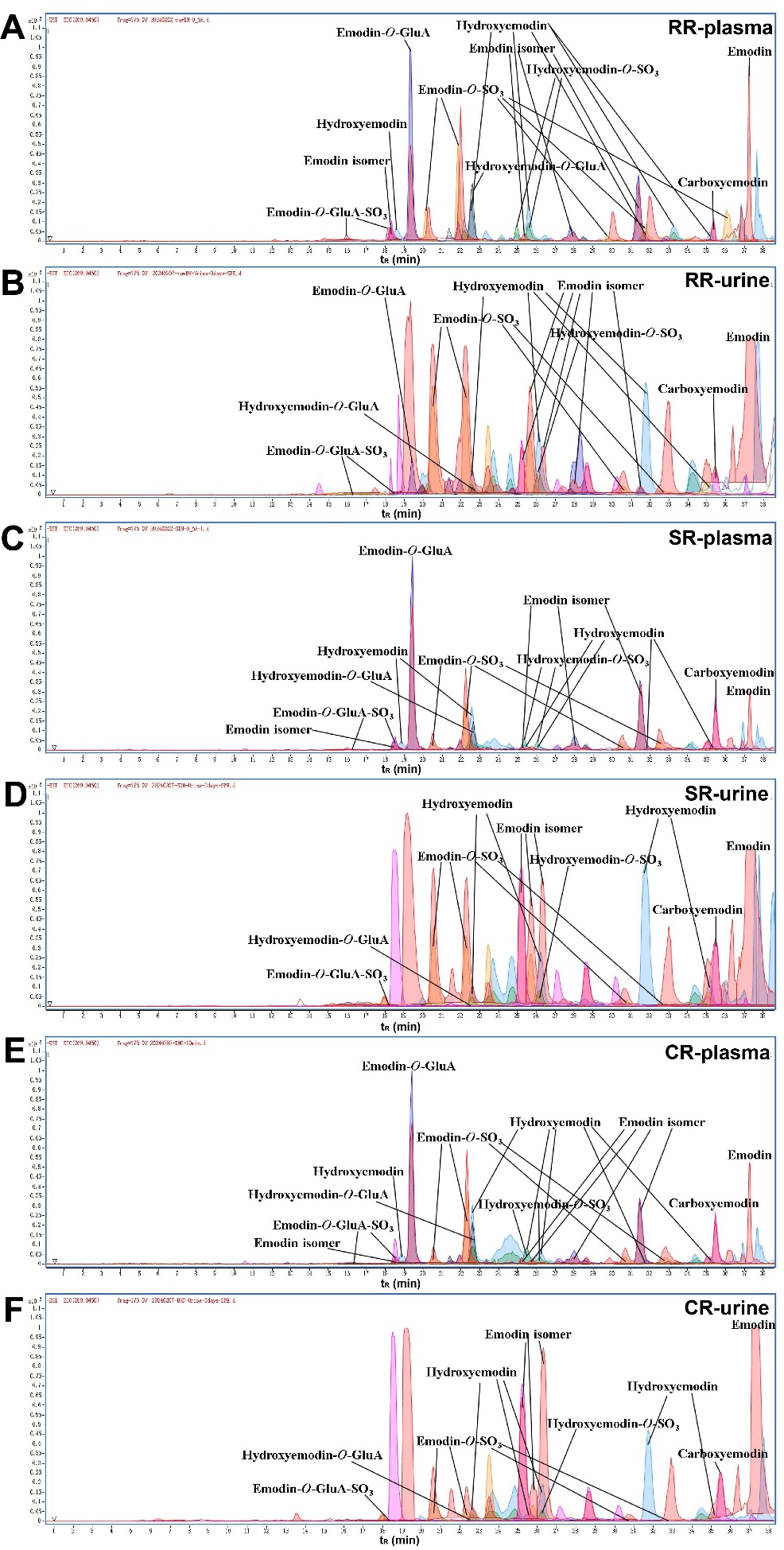


**Supplementary Figure 3.** The EICs of emodin and its metabolites in rats.


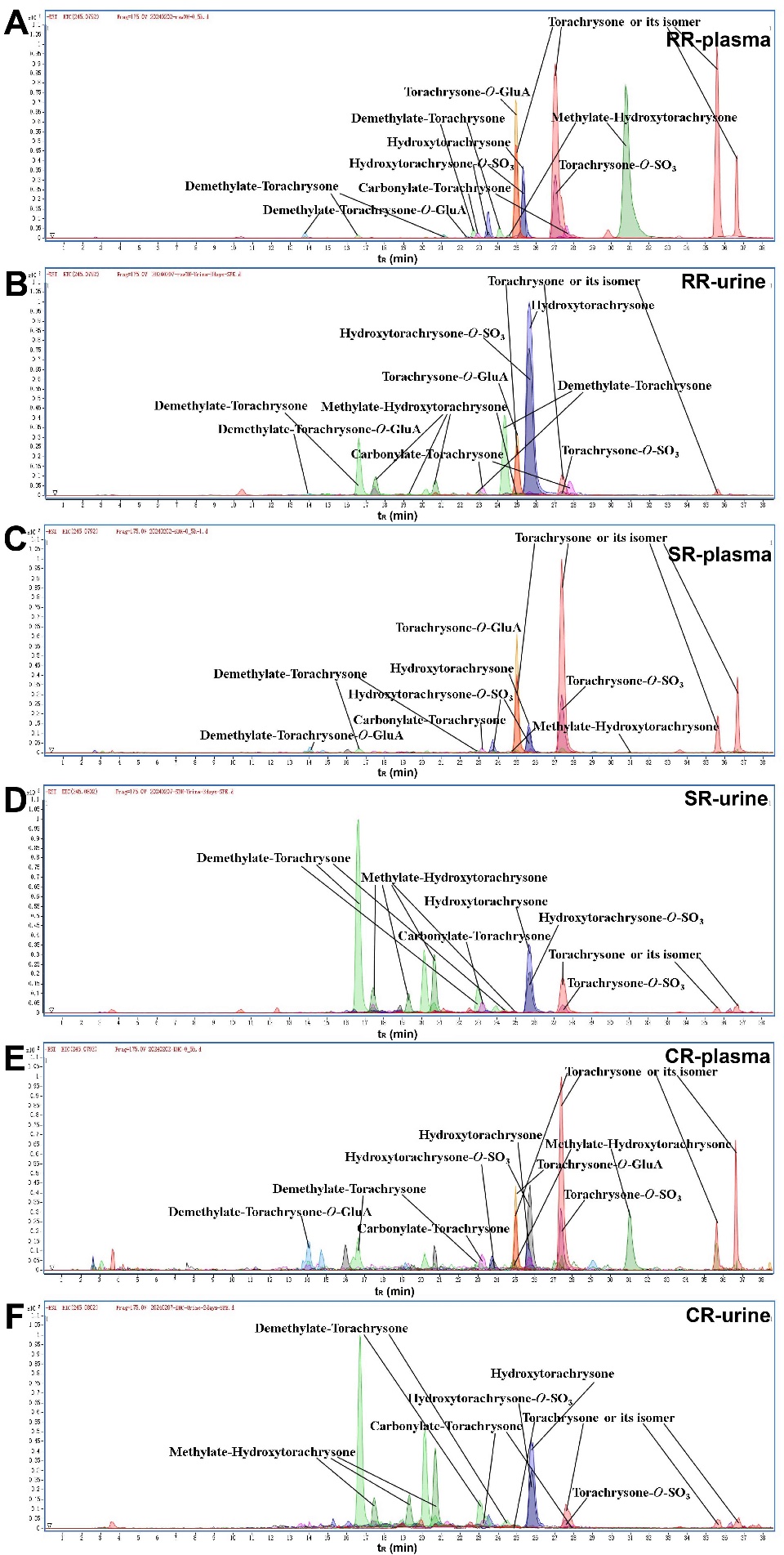


**Supplementary Figure 4.** The EICs of torachrysone and its metabolites in rats.


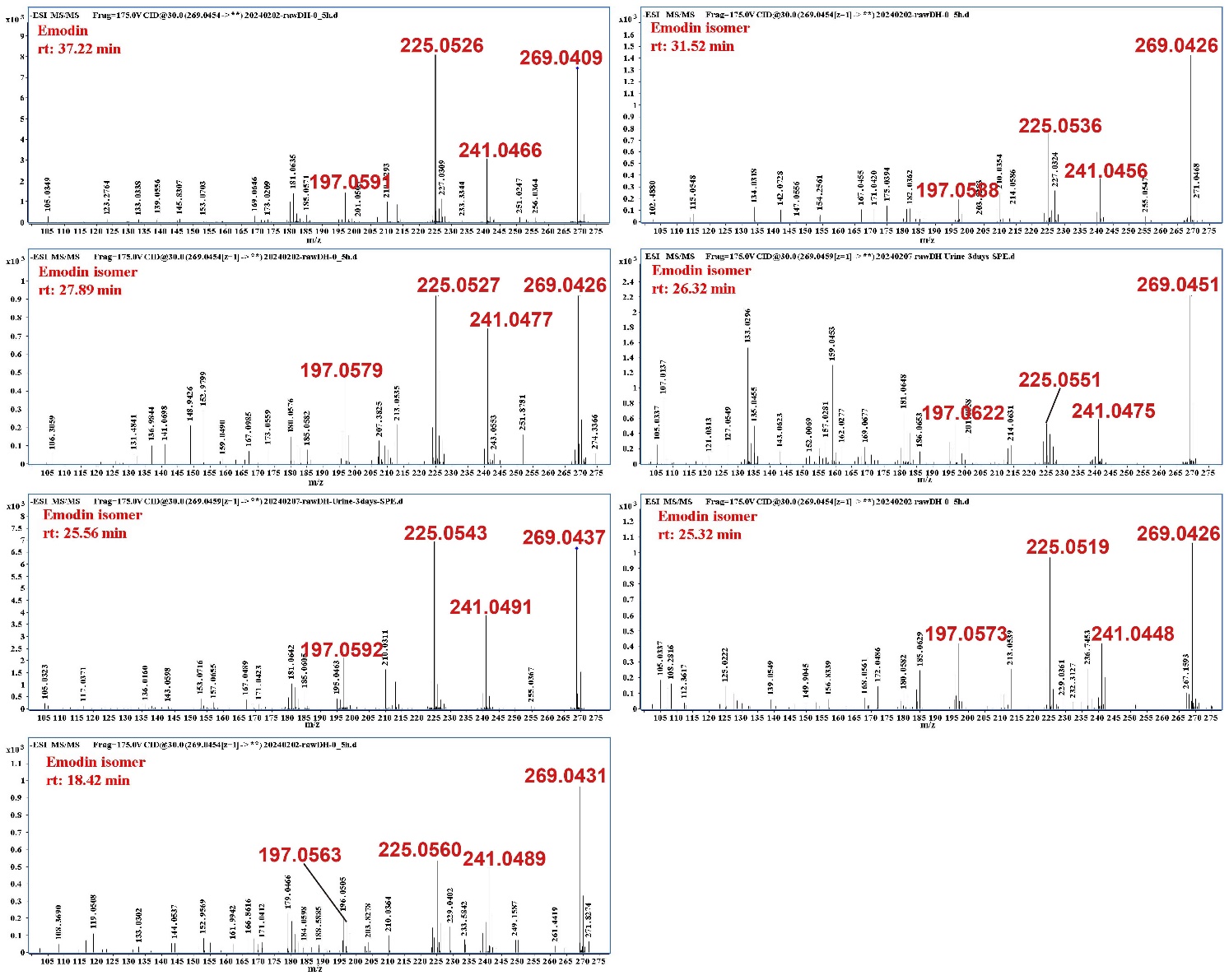


**Supplementary Figure 5.** The MS/MS spectra of emodin and its isomers.


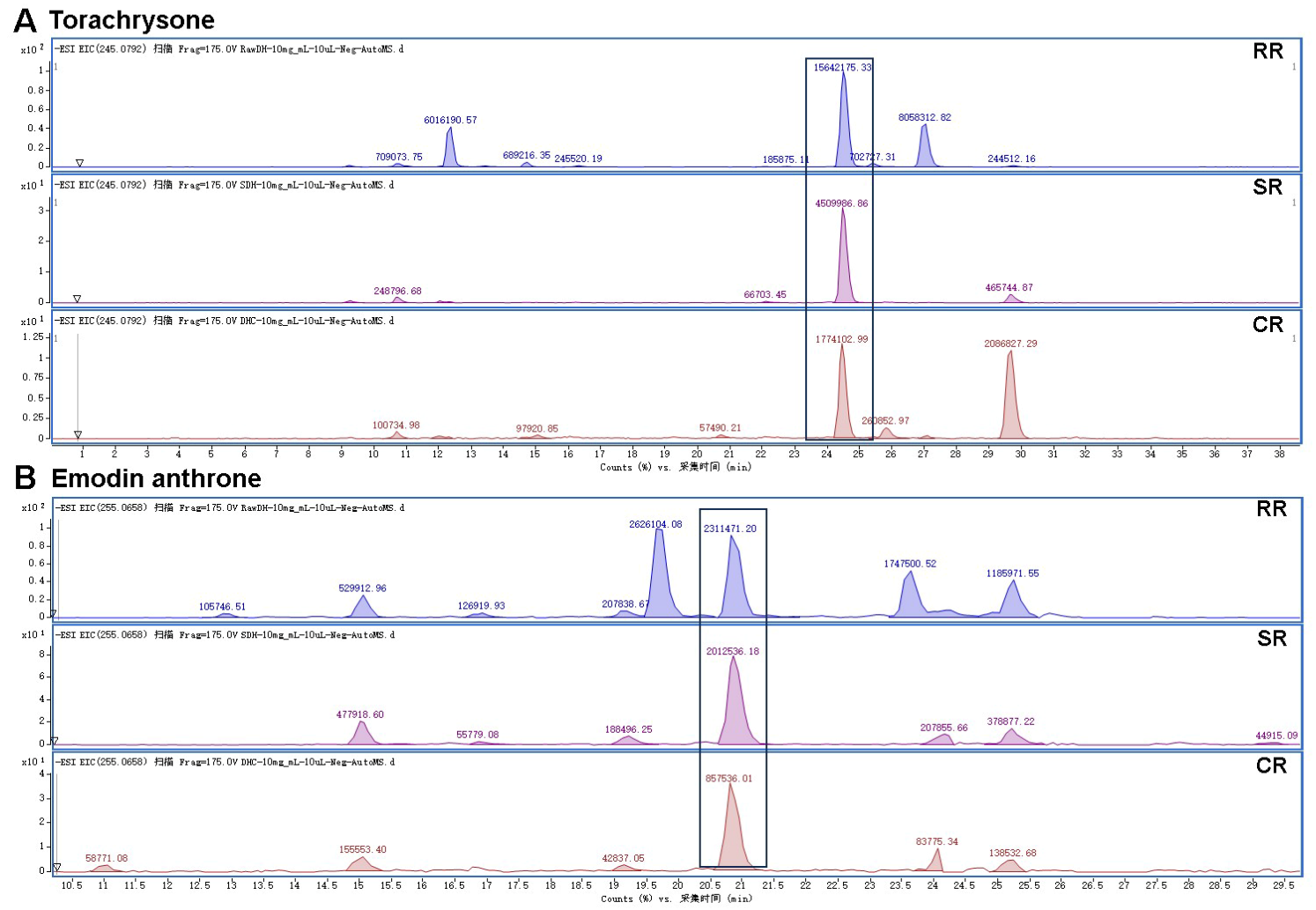


**Supplementary Figure 4.** Peak area comparison of (A) torachrysone and (B) emodin anthrone in three processed products of rhubarb.

## Supplementary Tables

**Supplementary Table 1** The identification results of compounds in RR, SR and CR.

| Num. | Name | Rt  (min) | *m/z*  (Neg) | Formula | MS/MS | RR | SR | CR | Sample |
| --- | --- | --- | --- | --- | --- | --- | --- | --- | --- |
| C1 | Ornithine | 2.84 | 131.0834 | C_5_H_12_N_2_O_2_ | 114.0550 | ＋ | ＋ | ＋ | Fr1 |
| C2 | Arginine | 2.84 | 173.1045 | C_6_H_14_N_4_O_2_ | 131.0838 | ＋ | ＋ | ＋ | Zong |
| C3 | Glutamine | 2.89 | 145.0621 | C_5_H_10_N_2_O_3_ | 127.0520, 109.0412 | ＋ | ＋ | ＋ | Zong |
| C4 | Histidine | 2.89 | 154.0621 | C_6_H_9_N_3_O_2_ | 137.0350, 110.0727 | ＋ | ＋ | - | Zong |
| C5 | *N*_2_-Fructopyranosylarginine | 2.89 | 335.1555 | C_12_H_24_N_4_O_7_ | 173.1031, 131.0830 | ＋ | ＋ | ＋ | Zong |
| C6 | Sucrose | 3.13 | 341.1139 | C_12_H_22_O_11_ | 179.0553, 161.0448, 101.0245 | ＋ | - | ＋ | Zong |
| C7 | Raffinose | 3.30 | 503.1625 | C_18_H_32_O_16_ | 323.0952, 179.0551 | ＋ | - | ＋ | Zong |
| C8 | Malic acid | 3.70 | 133.0137 | C_4_H_6_O_5_ | 115.0037 | ＋ | ＋ | ＋ | Zong |
| C9 | 2/6/1'/4'/6'-*O*-Galloylsucrose | 3.82 | 493.1188 | C_19_H_26_O_15_ | 331.0649, 169.0131, 125.0237 | ＋ | ＋ | ＋ | Zong |
| C10 | Galloylglucose | 3.99 | 331.0651 | C_13_H_16_O_10_ | 211.0243, 169.0139, 151.0036, 125.0244 | ＋ | ＋ | ＋ | Zong |
| C11 | Citramalic acid or its isomer | 4.52 | 147.0295 | C_5_H_8_O_5_ | 129.0189, 103.0405 | ＋ | ＋ | ＋ | Zong |
| C12 | Citric acid | 4.52 | 191.0191 | C_6_H_8_O_7_ | 111.0095 | ＋ | ＋ | ＋ | Zong |
| C13 | Galloylglucose | 4.87 | 331.0668 | C_13_H_16_O_10_ | 211.0238, 169.0129, 151.0033, 125.0237 | ＋ | ＋ | ＋ | Zong |
| C14 | 2/6/1'/4'/6'-*O*-Galloylsucrose | 5.16 | 493.1183 | C_19_H_26_O_15_ | 331.0660, 169.0124, 125.0242 | ＋ | ＋ | ＋ | Zong |
| C15 | Tyrosine | 5.16 | 180.0662 | C_9_H_11_NO_3_ | 163.0392, 119.0503 | ＋ | ＋ | ＋ | Zong |
| C16 | Citramalic acid or its isomer | 5.33 | 147.0296 | C_5_H_8_O_5_ | 129.0186, 103.0376 | ＋ | ＋ | - | Fr1 |
| C17 | Galloylglucose | 5.39 | 331.0655 | C_13_H_16_O_10_ | 211.0232, 169.0139, 151.0033, 125.0245 | ＋ | ＋ | ＋ | Zong |
| C18 | (Epi)Catechin trimers | 5.64 | 865.1871 | C_45_H_38_O_18_ | 739.1553, 713.1357, 695.1475, 577.1318, 451.0959, 407.0704, 287.0516 | ＋ | - | - | Zong |
| C19 | Hydroxyhydroquinone or Phloroglucinol or 1,2,3-Trihydroxybenzene | 6.42 | 125.0247 | C_6_H_6_O_3_ | 107.0133 | ＋ | ＋ | ＋ | Zong |
| C20 | Gallic acid | 7.02 | 169.0166 | C_7_H_6_O_5_ | 125.0247 | ＋ | ＋ | ＋ | Zong |
| C21 | Hydroxyhydroquinone or Phloroglucinol or 1,2,3-Trihydroxybenzene | 7.02 | 125.0283 | C_6_H_6_O_3_ | 107.0150 | ＋ | ＋ | ＋ | Zong |
| C22 | 2/6/1'/4'/6'-*O*-Galloylsucrose | 7.39 | 493.1161 | C_19_H_26_O_15_ | 331.0673, 169.0135, 125.0233 | ＋ | ＋ | ＋ | Zong |
| C23 | Phenylalanine | 7.78 | 164.0717 | C_9_H_11_NO_2_ | 147.0451, 103.0558 | ＋ | ＋ | ＋ | Zong |
| C24 | 2/6/1'/4'/6'-*O*-Galloylsucrose | 7.81 | 493.1173 | C_19_H_26_O_15_ | 331.0676, 169.0134, 125.0236 | ＋ | ＋ | ＋ | Zong |
| C25 | Methoxy-galloylglucose | 8.38 | 345.0797 | C_14_H_18_O_10_ | 183.0286, 168.0067 | ＋ | - | ＋ | Fr1 |
| C26 | Gallocatechin or its isomer | 8.63 | 305.0645 | C_15_H_14_O_7_ | 179.0337, 137.0261, 125.0235 | ＋ | - | - | Zong |
| C27 | 1,2-di-*O*-galloyl-*β*-D-glucose or 1,6-di-*O*-galloyl-*β*-D-glucose or its isomer | 8.96 | 483.0735 | C_20_H_20_O_14_ | 331.0626, 313.0546, 169.0137, 125.0248 | ＋ | - | - | Zong |
| C28 | Hydroxyhydroquinone or Phloroglucinol or 1,2,3-Trihydroxybenzene | 9.19 | 125.0247 | C_6_H_6_O_3_ | 107.0144 | ＋ | ＋ | ＋ | Zong |
| C29 | (+)-Catechin or Epicatechin or its isomer | 9.21 | 289.0708 | C_15_H_14_O_6_ | 271.0577, 245.0807, 205.0485, 179.0335, 125.0243 | ＋ | ＋ | ＋ | Zong |
| C30 | (Epi)Catechin glucoside | 9.21 | 451.1242 | C_21_H_24_O_11_ | 313.0691, 289.0707, 271.0629, 245.0780, 137.0246 | ＋ | ＋ | ＋ | Zong |
| C31 | Epicatechin-(4beta->8)-epicatechin-(4beta->8)-catechin 3/3'/3''-gallate | 9.39 | 1017.2032 | C_52_H_42_O_22_ | 577.1347, 407.0819, 289.0672, 125.0241 | ＋ | - | - | Zong |
| C32 | (Epi)Catechin dimers | 10.15 | 577.1374 | C_30_H_26_O_12_ | 451.1034, 425.0814, 289.0709, 245.0812, 125.0243 | ＋ | - | - | Zong |
| C33 | (Epi)Catechin trimers | 10.45 | 865.1879 | C_45_H_38_O_18_ | 739.1594, 713.1444, 695.1390, 577.1372, 451.0921, 407.0825, 287.0599 | ＋ | - | - | Zong |
| C34 | Methoxy-galloylglucose | 10.46 | 345.0804 | C_14_H_18_O_10_ | 183.0280, 168.0090 | ＋ | ＋ | ＋ | Fr1 |
| C35 | (Epi)Catechin dimers | 10.53 | 577.1379 | C_30_H_26_O_12_ | 451.0986, 425.0849, 289.0713, 245.0792, 125.0243 | ＋ | - | - | Zong |
| C36 | Coumaric acid glucoside | 10.62 | 325.0908 | C_15_H_18_O_8_ | 265.0657, 145.0296, 119.0495, 117.0339 | ＋ | ＋ | ＋ | Zong |
| C37 | Benzenedicarboxylic acid | 10.73 | 165.0187 | C_8_H_6_O_4_ | 121.0300 | ＋ | ＋ | ＋ | Fr2 |
| C38 | (+)-Catechin or Epicatechin or its isomer | 10.73 | 289.0717 | C_15_H_14_O_6_ | 271.0577, 245.0830, 205.0502, 179.0322, 125.0249 | ＋ | ＋ | ＋ | Zong |
| C39 | (Epi)Catechin glucoside | 10.77 | 451.1264 | C_21_H_24_O_11_ | 313.0694, 289.0702, 271.0649, 245.0811, 137.0244 | ＋ | ＋ | ＋ | Zong |
| C40 | 1,2-di-*O*-galloyl-*β*-D-glucose or 1,6-di-*O*-galloyl-*β*-D-glucose or its isomer | 10.78 | 483.0734 | C_20_H_20_O_14_ | 331.0689, 313.0556, 169.0163, 125.0230 | ＋ | - | - | Fr2 |
| C41 | Tryptophan | 11.17 | 203.0824 | C_11_H_12_N_2_O_2_ | 159.0939, 116.0512 | ＋ | ＋ | - | Zong |
| C42 | (Epi)Catechin trimers | 11.35 | 865.1872 | C_45_H_38_O_18_ | 739.1445, 713.1368, 695.1337, 577.1420, 451.0951, 407.0750, 287.0514 | ＋ | - | - | Zong |
| C43 | Epicatechin-(4beta->8)-epicatechin-(4beta->8)-catechin 3/3'/3''-gallate | 11.35 | 1017.1922 | C_52_H_42_O_22_ | 577.1265, 407.0762, 289.0731, 125.0259 | ＋ | - | - | Zong |
| C44 | Coumaric acid glucoside | 11.35 | 325.0914 | C_15_H_18_O_8_ | 265.0705, 145.0287, 119.0506, 117.0346 | ＋ | ＋ | ＋ | Zong |
| C45 | Methoxy-galloylglucose | 11.53 | 345.0804 | C_14_H_18_O_10_ | 183.0279, 168.0067 | ＋ | ＋ | ＋ | Fr1 |
| C46 | 1,2-di-*O*-galloyl-*β*-D-glucose or 1,6-di-*O*-galloyl-*β*-D-glucose or its isomer | 11.53 | 483.0728 | C_20_H_20_O_14_ | 331.0667, 313.0569, 169.0146, 125.0237 | ＋ | ＋ | - | Zong |
| C47 | Benzenedicarboxylic acid | 11.67 | 165.0185 | C_8_H_6_O_4_ | 121.0306 | ＋ | ＋ | ＋ | Fr2 |
| C48 | *O*-Methylgallic acid | 11.98 | 183.0297 | C_8_H_8_O_5_ | 168.0061, 139.0415, 124.0152 | ＋ | ＋ | ＋ | Zong |
| C49 | Epicatechin-(4beta->8)-epicatechin-(4beta->8)-catechin 3/3'/3''-gallate | 11.98 | 1017.2002 | C_52_H_42_O_22_ | 577.1299, 407.0715, 289.0677, 125.0257 | ＋ | - | - | Zong |
| C50 | (Epi)Catechin glucoside | 12.08 | 451.1264 | C_21_H_24_O_11_ | 313.0522, 289.0694, 271.0640, 245.1000, 137.0239 | ＋ | ＋ | ＋ | Zong |
| C51 | (Epi)Catechin dimers | 12.17 | 577.1318 | C_30_H_26_O_12_ | 451.0935, 425.0828, 289.0692, 245.0812, 125.0248 | ＋ | - | - | Zong |
| C52 | Benzenedicarboxylic acid | 12.32 | 165.0188 | C_8_H_6_O_4_ | 121.0303 | ＋ | ＋ | ＋ | Zong |
| C53 | (+)-Catechin or Epicatechin or its isomer | 12.32 | 289.0720 | C_15_H_14_O_6_ | 245.0810, 205.0508, 179.0349, 125.0236 | ＋ | ＋ | ＋ | Zong |
| C54 | Coumaric acid glucoside | 12.48 | 325.0912 | C_15_H_18_O_8_ | 265.0700, 145.0293, 119.0501, 117.0343 | ＋ | ＋ | ＋ | Zong |
| C55 | 6-hydroxymusizin-8-*O*-*β*-D-glucopyranoside or its isomer | 12.48 | 393.1155 | C_19_H_22_O_9_ | 231.0641, 189.0575 | ＋ | ＋ | - | Zong |
| C56 | 1,2-di-*O*-galloyl-*β*-D-glucose or 1,6-di-*O*-galloyl-*β*-D-glucose or its isomer | 12.53 | 483.0733 | C_20_H_20_O_14_ | 331.0772, 313.0599, 169.0139, 125.0240 | ＋ | - | ＋ | Zong |
| C57 | (Epi)Catechin trimers | 12.64 | 865.1877 | C_45_H_38_O_18_ | 739.1485, 713.1447, 695.1304, 577.1265, 451.1004, 407.0735, 287.0536 | ＋ | - | - | Zong |
| C58 | Methyl gallate | 12.82 | 183.0292 | C_8_H_8_O_5_ | 168.0093, 124.0152 | ＋ | ＋ | ＋ | Zong |
| C59 | Epicatechin-(4beta->8)-epicatechin-(4beta->8)-catechin 3/3'/3''-gallate | 12.89 | 1017.1964 | C_52_H_42_O_22_ | 577.1325, 407.0825, 289.0702, 125.0257 | ＋ | - | - | Zong |
| C60 | (Epi)Catechin dimers gallate | 12.89 | 729.1441 | C_37_H_30_O_16_ | 577.1292, 407.0729, 289.0699, 169.0133, 125.0248 | ＋ | - | - | Zong |
| C61 | Homogentisic acid | 13.02 | 167.0341 | C_8_H_8_O_4_ | 123.0447, 108.0239 | ＋ | ＋ | ＋ | Zong |
| C62 | Gallocatechin or its isomer | 13.12 | 305.0648 | C_15_H_14_O_7_ | 179.0323, 137.0246, 125.0249 | ＋ | - | - | Zong |
| C63 | Epicatechin-(4beta->8)-epicatechin-(4beta->8)-catechin 3/3'/3''-gallate | 13.25 | 1017.1967 | C_52_H_42_O_22_ | 577.1340, 407.0792, 289.0681, 125.0239 | ＋ | - | - | Zong |
| C64 | (Epi)Catechin dimers gallate | 13.25 | 729.1438 | C_37_H_30_O_16_ | 577.1340, 407.0729, 289.0699, 169.0133, 125.0248 | ＋ | - | - | Zong |
| C65 | Coumaric acid glucoside | 13.25 | 325.0920 | C_15_H_18_O_8_ | 265.0684, 145.0296, 119.0508, 117.0349 | ＋ | ＋ | ＋ | Zong |
| C66 | (+)-Catechin or Epicatechin or its isomer | 13.46 | 289.0712 | C_15_H_14_O_6_ | 245.0800, 205.0503, 179.0340, 125.0233 | ＋ | - | - | Zong |
| C67 | (Epi)Catechin dimers | 13.76 | 577.1325 | C_30_H_26_O_12_ | 451.1089, 425.0858, 289.0723, 245.0806, 125.0237 | ＋ | - | - | Zong |
| C68 | 6-hydroxymusizin-8-*O*-*β*-D-glucopyranoside or its isomer | 13.76 | 393.1169 | C_19_H_22_O_9_ | 231.0644, 189.0537 | ＋ | ＋ | ＋ | Zong |
| C69 | 1,2-di-*O*-galloyl-*β*-D-glucose or 1,6-di-*O*-galloyl-*β*-D-glucose or its isomer | 13.97 | 483.0736 | C_20_H_20_O_14_ | 331.0650, 313.0537, 169.0129, 125.0224 | ＋ | ＋ | ＋ | Zong |
| C70 | Tri-*O*-galloyl-glucose | 13.97 | 635.0874 | C_27_H_24_O_18_ | 465.0617, 313.0573, 169.0129 | ＋ | - | - | Zong |
| C71 | (Epi)Catechin dimers gallate | 14.12 | 729.1423 | C_37_H_30_O_16_ | 577.1300, 407.0776, 289.0687, 169.0127, 125.0228 | ＋ | - | - | Zong |
| C72 | Epicatechin-(4beta->8)-epicatechin-(4beta->8)-catechin 3/3'/3''-gallate | 14.21 | 1017.1964 | C_52_H_42_O_22_ | 577.1325, 407.0692, 289.0680, 125.0248 | ＋ | - | - | Zong |
| C73 | Procyanidin B2 3,3'-di-*O*-gallate | 14.21 | 881.1517 | C_44_H_34_O_20_ | 729.1448, 559.1256, 541.1026, 407.0750, 289.0695, 169.0145, 125.0245 | ＋ | - | - | Zong |
| C74 | Coumaric acid glucoside | 14.29 | 325.0920 | C_15_H_18_O_8_ | 265.0704, 145.0295, 119.0495, 117.0348 | ＋ | ＋ | ＋ | Zong |
| C75 | (Epi)Catechin trimers | 14.45 | 865.1883 | C_45_H_38_O_18_ | 739.1574, 713.1353, 695.1352, 577.1237, 451.1009, 407.0740, 287.0525 | ＋ | ＋ | - | Fr2 |
| C76 | Methyl gallate | 14.50 | 183.0300 | C_8_H_8_O_5_ | 168.0066, 124.0158 | ＋ | ＋ | ＋ | Fr2 |
| C77 | (+)-Catechin or Epicatechin or its isomer | 14.68 | 289.0729 | C_15_H_14_O_6_ | 245.0803, 205.0483, 179.0329, 125.0238 | ＋ | ＋ | ＋ | Zong |
| C78 | Epicatechin-(4beta->8)-epicatechin-(4beta->8)-catechin 3/3'/3''-gallate | 14.90 | 1017.1967 | C_52_H_42_O_22_ | 577.1382, 407.0738, 289.0699, 125.0235 | ＋ | - | - | Zong |
| C79 | Caffeic acid | 14.95 | 179.0345 | C_9_H_8_O_4_ | 135.0454, 107.0505 | ＋ | ＋ | ＋ | Zong |
| C80 | Phlorizin | 13.90 | 435.1217 | C_21_H_24_O_10_ | 273.0754, 167.0322 | ＋ | ＋ | - | Zong |
| C81 | Phlorizin isomer | 14.50 | 435.1258 | C_21_H_24_O_10_ | 273.0749, 167.0325 | ＋ | ＋ | ＋ | Fr1 |
| C82 | 2-(2'-hydroxypropyl)-5-methyl-7-hydroxychromone or its isomer | 15.15 | 233.0805 | C_13_H_14_O_4_ | 189.0542, 149.0228, 105.0348 | ＋ | ＋ | ＋ | Zong |
| C83 | (Epi)Catechin dimers gallate | 15.32 | 729.1385 | C_37_H_30_O_16_ | 577.1268, 407.0727, 289.0660, 169.0137, 125.0249 | ＋ | - | - | Zong |
| C84 | 6-*O*-galloyl-1-*O*-*p*-coumaroyl-*β*-D-glucose or 1-*O*-galloyl-6-*O*-*p*-coumaroyl-*β*-D-glucose or its isomer | 15.32 | 477.1033 | C_22_H_22_O_12_ | 313.0543, 169.0137, 125.0236 | ＋ | ＋ | ＋ | Zong |
| C85 | Coumaric acid glucoside | 15.32 | 325.0906 | C_15_H_18_O_8_ | 265.0708, 145.0281, 119.0516, 117.0347 | ＋ | ＋ | ＋ | Zong |
| C86 | Epicatechin-3-*O*-gallate or 7-Galloylcatechin or their isomer | 15.51 | 441.0839 | C_22_H_18_O_10_ | 289.0702, 271.0599, 169.0139, 125.0240 | ＋ | - | - | Fr2 |
| C87 | 6-*O*-galloyl-1-*O*-*p*-coumaroyl-*β*-D-glucose or 1-*O*-galloyl-6-*O*-*p*-coumaroyl-*β*-D-glucose or its isomer | 15.73 | 477.1020 | C_22_H_22_O_12_ | 313.0664, 169.0138, 125.0241 | ＋ | ＋ | ＋ | Zong |
| C88 | (Epi)Catechin glucoside | 15.92 | 451.1194 | C_21_H_24_O_11_ | 313.0543, 289.0784, 271.0459, 245.0806, 137.0244 | ＋ | ＋ | ＋ | Fr2 |
| C89 | Tri-*O*-galloyl-glucose | 16.16 | 635.0830 | C_27_H_24_O_18_ | 465.0613, 313.0620, 169.0152 | ＋ | - | - | Fr2 |
| C90 | (+)-Catechin or Epicatechin or its isomer | 16.34 | 289.0703 | C_15_H_14_O_6_ | 271.0571, 245.0795, 205.0505, 179.0340, 125.0246 | ＋ | - | - | Zong |
| C91 | Epicatechin-3-*O*-gallate or 7-Galloylcatechin or their isomer | 16.34 | 441.0814 | C_22_H_18_O_10_ | 289.0711, 271.0599, 169.0106, 125.0274 | ＋ | ＋ | ＋ | Zong |
| C92 | 6-hydroxymusizin-8-*O*-*β*-D-glucopyranoside or its isomer | 16.42 | 393.1148 | C_19_H_22_O_9_ | 231.0656, 189.0536 | ＋ | - | - | Zong |
| C93 | Emodin 1-*O*-*β*-D-glucopyranoside isomer or Emodin 8-*O*-*β*-D-glucopyranoside isomer | 16.85 | 431.0960 | C_21_H_20_O_10_ | 269.0436, 225.0527 | ＋ | ＋ | ＋ | Fr2 |
| C94 | Quercetin or its isomer | 16.95 | 301.0339 | C_15_H_10_O_7_ | 151.004, 121.0309 | ＋ | ＋ | ＋ | Fr3 |
| C95 | Lindleyin | 17.22 | 477.1383 | C_23_H_26_O_11_ | 313.0540, 169.0141, 125.0242 | ＋ | ＋ | ＋ | Zong |
| C96 | 6-*O*-galloyl-1-*O*-*p*-coumaroyl-*β*-D-glucose or 1-*O*-galloyl-6-*O*-*p*-coumaroyl-*β*-D-glucose or its isomer | 17.22 | 477.1020 | C_22_H_22_O_12_ | 313.0530, 169.0132, 125.0229 | ＋ | ＋ | ＋ | Zong |
| C97 | Epicatechin-3-O-gallate or 7-Galloylcatechin or their isomer | 17.33 | 441.0790 | C_22_H_18_O_10_ | 289.0698, 271.0589, 169.0133, 125.0249 | ＋ | ＋ | ＋ | Zong |
| C98 | Cinnamic acid | 17.33 | 147.0451 | C_9_H_8_O_2_ | 103.0556 | ＋ | ＋ | ＋ | Zong |
| C99 | Coumalic acid | 17.87 | 163.0398 | C_9_H_8_O_3_ | 119.0503 | ＋ | ＋ | ＋ | Zong |
| C100 | Chrysophanol-*O*-glucopyranoside | 17.87 | 415.1016 | C_21_H_20_O_9_ | 253.0495, 225.0529 | ＋ | ＋ | - | Zong |
| C101 | 2-(2'-hydroxypropyl)-5-methyl-7-hydroxychromone or its isomer | 18.01 | 233.0808 | C_13_H_14_O_4_ | 189.0562, 149.0241, 105.0334 | ＋ | ＋ | ＋ | Fr2 |
| C102 | 6-*O*-galloyl-1-*O*-*p*-coumaroyl-*β*-D-glucose or 1-*O*-galloyl-6-*O*-*p*-coumaroyl-*β*-D-glucose or its isomer | 18.01 | 477.1017 | C_22_H_22_O_12_ | 313.0574, 169.0132, 125.0246 | ＋ | ＋ | ＋ | Fr2 |
| C103 | 1,2-di-*O*-galloyl-6-*O*-*p*-coumaroyl-*β*-D-glucose or 1,6-di-*O*-galloyl-2-*O*-*p*-coumaroyl-*β*-D-glucose or 2,6-di-*O*-galloyl-1-*O*-*p*-coumaroyl-*β*-D-glucose or its isomer | 18.04 | 629.1118 | C_29_H_26_O_16_ | 313.0548, 169.0145, 125.0254 | ＋ | - | - | Zong |
| C104 | 1,2-di-*O*-galloyl-6-*O*-*p*-coumaroyl-*β*-D-glucose or 1,6-di-*O*-galloyl-2-*O*-*p*-coumaroyl-*β*-D-glucose or 2,6-di-*O*-galloyl-1-*O*-*p*-coumaroyl-*β*-D-glucose or its isomer | 18.44 | 629.1071 | C_29_H_26_O_16_ | 313.0573, 169.0126, 125.0247 | ＋ | - | - | Zong |
| C105 | Epicatechin-3-*O*-gallate or 7-Galloylcatechin or their isomer | 18.64 | 441.0791 | C_22_H_18_O_10_ | 289.0688, 271.0584, 169.0188, 125.0251 | ＋ | - | - | Fr2 |
| C106 | 6-*O*-galloyl-1-*O*-*p*-coumaroyl-*β*-D-glucose or 1-*O*-galloyl-6-*O*-*p*-coumaroyl-*β*-D-glucose or its isomer | 18.73 | 477.0992 | C_22_H_22_O_12_ | 313.0520, 169.0137, 125.0239 | ＋ | ＋ | ＋ | Zong |
| C107 | Aloe-emodin 8-*O*-*β*-D-glucopyranoside | 19.23 | 431.0971 | C_21_H_20_O_10_ | 269.0417, 240.0410 | ＋ | ＋ | ＋ | Zong |
| C108 | Aloe-emodin isomer | 19.23 | 269.0442 | C_15_H_10_O_5_ | 239.0337 | ＋ | ＋ | ＋ | Zong |
| C109 | 1-*O*-galloyl-6-*O*-cinnamoyl-*β*-D-glucose or 2-*O*-galloyl-1-*O*-cinnamoyl-*β*-D-glucose or 1-*O*-galloyl-2-*O*-cinnamoyl-*β*-D-glucose | 19.39 | 461.1094 | C_22_H_22_O_11_ | 313.0538, 271.0453, 211.0230, 169.0134, 147.0444 | ＋ | ＋ | ＋ | Zong |
| C110 | Sennoside C or D or their isomer | 19.50 | 847.2058 | C_42_H_40_O_19_ | 685.1526, 431.0979, 386.0999, 269.0482, 224.0453 | ＋ | - | - | Fr2 |
| C111 | Stilbene | 19.65 | 417.1154 | C_21_H_22_O_9_ | 255.0651, 227.0676 | ＋ | - | - | Zong |
| C112 | Emodin Anthrone or its isomer | 19.71 | 255.0655 | C_15_H_12_O_4_ | 213.0556, 171.0423, 145.0660 | ＋ | - | - | Zong |
| C113 | 1,2-di-*O*-galloyl-6-*O*-*p*-coumaroyl-*β*-D-glucose or 1,6-di-*O*-galloyl-2-*O*-*p*-coumaroyl-*β*-D-glucose or 2,6-di-*O*-galloyl-1-*O*-*p*-coumaroyl-*β*-D-glucose or its isomer | 19.74 | 629.1113 | C_29_H_26_O_16_ | 313.0520, 169.0135, 125.0243 | ＋ | ＋ | - | Zong |
| C114 | Sennoside C or D or their isomer | 19.86 | 847.2061 | C_42_H_40_O_19_ | 685.1517, 431.0882, 386.1006, 269.0404, 224.0417 | ＋ | - | - | Zong |
| C115 | Sennoside C or D or their isomer | 20.37 | 847.2040 | C_42_H_40_O_19_ | 685.1512, 431.0898, 386.0988, 269.0426, 224.0462 | ＋ | - | - | Zong |
| C116 | 1,2-di-*O*-galloyl-6-*O*-*p*-coumaroyl-*β*-D-glucose or 1,6-di-*O*-galloyl-2-*O*-*p*-coumaroyl-*β*-D-glucose or 2,6-di-*O*-galloyl-1-*O*-*p*-coumaroyl-*β*-D-glucose or its isomer | 20.46 | 629.1070 | C_29_H_26_O_16_ | 313.0594, 169.0144, 125.0240 | ＋ | - | - | Fr2 |
| C117 | Chrysophanol-*O*-glucopyranoside | 20.59 | 415.1015 | C_21_H_20_O_9_ | 253.0494, 225.0561 | ＋ | ＋ | - | Zong |
| C118 | Quercetin or its isomer | 20.75 | 301.0344 | C_15_H_10_O_7_ | 151.0057, 121.0284 | ＋ | - | - | Fr3 |
| C119 | Aloe-emodin 1-*O*-*β*-D-glucopyranoside | 20.83 | 431.0988 | C_21_H_20_O_10_ | 269.0442, 240.0407 | ＋ | ＋ | ＋ | Fr2 |
| C120 | Sennoside B | 20.83 | 861.1853 | C_42_H_38_O_20_ | 699.1248, 386.0975, 224.0459 | ＋ | - | - | Zong |
| C121 | Stilbene | 20.83 | 417.1154 | C_21_H_22_O_9_ | 255.0656, 227.0684 | ＋ | ＋ | ＋ | Zong |
| C122 | Emodin Anthrone or its isomer | 20.94 | 255.0658 | C_15_H_12_O_4_ | 213.0554, 171.0463, 145.0657 | ＋ | ＋ | ＋ | Zong |
| C123 | 1-*O*-galloyl-6-*O*-cinnamoyl-*β*-D-glucose or 2-*O*-galloyl-1-*O*-cinnamoyl-*β*-D-glucose or 1-*O*-galloyl-2-*O*-cinnamoyl-*β*-D-glucose | 21.06 | 461.1098 | C_22_H_22_O_11_ | 313.0567, 271.0424, 211.0266, 169.0142, 147.0448 | ＋ | - | ＋ | Zong |
| C124 | 6-hydroxymusizin-8-*O*-*β*-D-glucopyranoside or its isomer | 21.18 | 393.1156 | C_19_H_22_O_9_ | 231.0651, 189.0517 | ＋ | ＋ | ＋ | Zong |
| C125 | Sennoside C or D or their isomer | 21.29 | 847.2040 | C_42_H_40_O_19_ | 685.1512, 431.0851, 386.0985, 269.0406, 224.0465 | ＋ | - | - | Zong |
| C126 | Chrysazin | 21.40 | 239.0343 | C_14_H_8_O_4_ | 211.0383, 183.0449 | ＋ | ＋ | ＋ | Zong |
| C127 | Rhein isomer | 21.47 | 283.0234 | C_15_H_8_O_6_ | 239.0310, 211.0377, 183.0436 | ＋ | ＋ | ＋ | Zong |
| C128 | Rhein 8-*O*-*β*-D-glucopyranoside | 21.47 | 445.0767 | C_21_H_18_O_11_ | 283.0247, 239.0338, 211.0373, 183.0461 | ＋ | ＋ | ＋ | Zong |
| C129 | 1,6-di-*O*-galloyl-2-*O*-cinnamoyl-*β*-D-glucose or 1,2-di-*O*-galloyl-6-*O*-cinnamoyl-*β*-D-glucose or 2,6-di-*O*-galloyl-1-*O*-cinnamoyl-*β*-D-glucose | 21.67 | 613.1170 | C_29_H_26_O_15_ | 465.0695, 313.0634, 211.0245, 169.0148 | ＋ | ＋ | ＋ | Zong |
| C130 | 2-(2'-hydroxypropyl)-5-methyl-7-hydroxychromone or its isomer | 21.82 | 233.0808 | C_13_H_14_O_4_ | 215.0676, 189.0568, 149.0238, 105.0332 | ＋ | ＋ | ＋ | Zong |
| C131 | Aloe-emodin 1-*O*-*β*-D-glucopyranoside isomer or Aloe-emodin 8-*O*-*β*-D-glucopyranoside isomer | 21.86 | 431.0939 | C_21_H_20_O_10_ | 269.0450, 240.0411 | ＋ | ＋ | ＋ | Fr2 |
| C132 | 1,2-di-*O*-galloyl-6-*O*-*p*-coumaroyl-*β*-D-glucose or 1,6-di-*O*-galloyl-2-*O*-*p*-coumaroyl-*β*-D-glucose or 2,6-di-*O*-galloyl-1-*O*-*p*-coumaroyl-*β*-D-glucose or its isomer | 22.10 | 629.1074 | C_29_H_26_O_16_ | 313.0552, 169.0140, 125.0245 | ＋ | ＋ | ＋ | Zong |
| C133 | 1,6-di-*O*-galloyl-2-*O*-cinnamoyl-*β*-D-glucose or 1,2-di-*O*-galloyl-6-*O*-cinnamoyl-*β*-D-glucose or 2,6-di-*O*-galloyl-1-*O*-cinnamoyl-β-D-glucose | 22.10 | 613.1167 | C_29_H_26_O_15_ | 465.0652, 313.0578, 211.0222, 169.0123 | ＋ | ＋ | ＋ | Zong |
| C134 | Emodin 8-*O*-*β*-D-(6’-*O*-Malonylglucoside) or Emodin 1-*O*-*β*-D-(6’-*O*-Malonylglucoside) | 22.20 | 517.0933 | C_24_H_22_O_13_ | 473.1058, 311.0549, 269.0406, 225.0553 | ＋ | ＋ | - | Zong |
| C135 | 1-*O*-galloyl-6-*O*-cinnamoyl-*β*-D-glucose or 2-*O*-galloyl-1-*O*-cinnamoyl-*β*-D-glucose or 1-*O*-galloyl-2-O-cinnamoyl-*β*-D-glucose | 22.54 | 461.1144 | C_22_H_22_O_11_ | 313.0550, 271.0447, 211.0243, 169.0136, 147.0448 | ＋ | ＋ | ＋ | Zong |
| C136 | Sennoside A | 22.83 | 861.1850 | C_42_H_38_O_20_ | 699.1333, 386.0977, 224.0463 | ＋ | - | - | Zong |
| C137 | 1,6-di-*O*-galloyl-2-*O*-cinnamoyl-*β*-D-glucose or 1,2-di-*O*-galloyl-6-*O*-cinnamoyl-*β*-D-glucose or 2,6-di-*O*-galloyl-1-*O*-cinnamoyl-*β*-D-glucose | 23.31 | 613.1131 | C_29_H_26_O_15_ | 313.0623, 211.0240, 169.0130 | ＋ | - | ＋ | Zong |
| C138 | 5, 5'-bis(*β*-D-glucopyranosyloxy) rheidin B or its isomer | 23.41 | 831.2031 | C_42_H_40_O_18_ | 669.1582, 386.0952, 269.0454, 224.0500 | ＋ | - | - | Zong |
| C139 | Stilbene | 23.61 | 417.1150 | C_21_H_22_O_9_ | 255.0643, 227.0727 | ＋ | - | - | Zong |
| C140 | Emodin 1-*O*-*β*-D-glucopyranoside | 24.22 | 431.1006 | C_21_H_20_O_10_ | 269.0446, 225.0560 | ＋ | ＋ | ＋ | Zong |
| C141 | 5, 5'-bis(*β*-D-glucopyranosyloxy) rheidin B or its isomer | 24.22 | 831.2097 | C_42_H_40_O_18_ | 669.1501, 386.1000, 269.0426, 224.0465 | ＋ | - | - | Zong |
| C142 | Rhein isomer | 24.35 | 283.0237 | C_15_H_8_O_6_ | 239.0351, 211.0389, 183.0440 | ＋ | - | - | Zong |
| C143 | Rhein derivative | 24.35 | 487.0827 | C_23_H_20_O_12_ | 283.0221, 239.0324 | ＋ | - | - | Fr2 |
| C144 | Torachrysone or its isomer | 24.49 | 245.0833 | C_14_H_14_O_4_ | 230.0572, 215.0331, 159.0433 | ＋ | ＋ | ＋ | Zong |
| C145 | Torachrysone 8-*O*-*β*-D-glucopyranoside | 24.49 | 407.1381 | C_20_H_24_O_9_ | 245.0794, 230.0575, 215.0394, 159.0450, 131.0499 | ＋ | ＋ | ＋ | Zong |
| C146 | Resveratrol 4'-*O*-*β*-D-glucopyranoside | 24.79 | 389.1216 | C_20_H_22_O_8_ | 227.0711, 185.0654 | ＋ | - | - | Zong |
| C147 | Quercetin or its isomer | 25.03 | 301.0355 | C_15_H_10_O_7_ | 178.9996, 151.0034, 121.0282 | ＋ | ＋ | ＋ | Zong |
| C148 | Stilbene | 25.41 | 449.1407 | C_22_H_25_O_10_ | 245.0794 | ＋ | ＋ | ＋ | Zong |
| C149 | Torachrysone or its isomer | 25.42 | 245.0800 | C_14_H_14_O_4_ | 230.0547, 215.0341, 159.0435 | ＋ | - | - | Zong |
| C150 | 5, 5'-bis(*β*-D-glucopyranosyloxy) rheidin B or its isomer | 25.42 | 831.2106 | C_42_H_40_O_18_ | 669.1493, 386.0922, 269.0427, 224.0466 | ＋ | - | - | Zong |
| C151 | Chrysophanol isomer | 25.59 | 253.0499 | C_15_H_10_O_4_ | 225.0567 | ＋ | - | - | Fr2 |
| C152 | 5, 5'-bis(*β*-D-glucopyranosyloxy) rheidin B or its isomer | 26.00 | 831.2014 | C_42_H_40_O_18_ | 669.1499, 386.0965, 269.0453, 224.0454 | ＋ | - | - | Zong |
| C153 | Rhein derivative | 26.08 | 487.0828 | C_23_H_20_O_12_ | 283.0229, 239.0337 | ＋ | ＋ | ＋ | Zong |
| C154 | Chrysophanol-*O*-glucopyranoside | 26.49 | 415.1028 | C_21_H_20_O_9_ | 253.0501, 225.0550 | ＋ | ＋ | ＋ | Zong |
| C155 | Chrysophanol isomer | 26.53 | 253.0500 | C_15_H_10_O_4_ | 225.0556 | ＋ | ＋ | ＋ | Zong |
| C156 | Stilbene | 26.93 | 449.1420 | C_22_H_25_O_10_ | 245.0806 | ＋ | ＋ | ＋ | Fr3 |
| C157 | Torachrysone or its isomer | 26.99 | 245.0810 | C_14_H_14_O_4_ | 230.0576, 215.0339, 159.0452 | ＋ | ＋ | ＋ | Zong |
| C158 | Chrysophanol-*O*-glucopyranoside | 27.04 | 415.1015 | C_21_H_20_O_9_ | 253.0500, 225.0561 | ＋ | ＋ | ＋ | Zong |
| C159 | Chrysophanol isomer | 27.05 | 253.0502 | C_15_H_10_O_4_ | 225.0553 | ＋ | ＋ | ＋ | Zong |
| C160 | Torachrysone or its isomer | 27.50 | 245.0810 | C_14_H_14_O_4_ | 230.0582, 215.0352, 159.0451 | ＋ | - | - | Fr2 |
| C161 | Emodin 8-*O*-*β*-D-glucopyranoside | 27.71 | 431.1004 | C_21_H_20_O_10_ | 269.0432, 225.0553 | ＋ | ＋ | ＋ | Zong |
| C162 | Emodin isomer | 27.71 | 269.0448 | C_15_H_10_O_5_ | 225.0550 | ＋ | ＋ | ＋ | Fr3 |
| C163 | Chrysophanol isomer | 28.33 | 253.0497 | C_15_H_10_O_4_ | 225.0531 | ＋ | ＋ | ＋ | Fr3 |
| C164 | Chrysophanol isomer | 29.14 | 253.0513 | C_15_H_10_O_4_ | 225.0551 | ＋ | ＋ | ＋ | Fr3 |
| C165 | Chrysophanol | 29.59 | 253.0502 | C_15_H_10_O_4_ | 225.054 | ＋ | ＋ | ＋ | Fr3 |
| C166 | Emodin 8-*O*-*β*-D-(6’-O-Malonylglucoside) or Emodin 1-*O*-*β*-D-(6’-O-Malonylglucoside) | 29.66 | 517.0912 | C_24_H_22_O_13_ | 473.1048, 311.0545, 269.0436, 225.0540 | ＋ | - | - | Zong |
| C167 | Stilbene | 29.69 | 449.1407 | C_22_H_25_O_10_ | 245.0804 | ＋ | ＋ | ＋ | Zong |
| C168 | Physcion isomer | 29.81 | 283.0610 | C_16_H_12_O_5_ | 240.0416 | ＋ | ＋ | ＋ | Zong |
| C169 | Physcion 8-*O*-*β*-D-glucopyranoside or its isomer | 29.84 | 445.1119 | C_22_H_22_O_10_ | 283.0591, 240.0409 | ＋ | ＋ | ＋ | Zong |
| C170 | Physcion | 30.79 | 283.0605 | C_16_H_12_O_5_ | 240.0428 | ＋ | ＋ | ＋ | Zong |
| C171 | Physcion 8-*O*-*β*-D-glucopyranoside or its isomer | 30.85 | 445.1121 | C_22_H_22_O_10_ | 283.0596, 240.0401 | ＋ | ＋ | ＋ | Zong |
| C172 | Emodin 8-*O*-*β*-D-glucopyranoside | 31.06 | 431.0941 | C_21_H_20_O_10_ | 269.0424, 225.0543 | ＋ | ＋ | ＋ | Fr3 |
| C173 | Emodin isomer | 31.06 | 269.0468 | C_15_H_10_O_5_ | 225.0533 | ＋ | ＋ | ＋ | Fr3 |
| C174 | Citreorosein | 31.66 | 285.0400 | C_15_H_10_O_6_ | 241.0445, 211.0353 | ＋ | ＋ | ＋ | Fr3 |
| C175 | Physcion isomer | 32.39 | 283.0595 | C_16_H_12_O_5_ | 240.0414 | ＋ | - | - | Fr3 |
| C176 | Aloe-emodin | 32.76 | 269.0438 | C_15_H_10_O_5_ | 239.032 | ＋ | ＋ | ＋ | Zong |
| C177 | Chrysophanol isomer | 32.87 | 253.0508 | C_15_H_10_O_4_ | 225.0577 | ＋ | ＋ | ＋ | Fr3 |
| C178 | 6-Methylrhein | 32.98 | 297.0399 | C_16_H_10_O_6_ | 253.0493, 225.0516 | ＋ | ＋ | ＋ | Zong |
| C179 | Physcion isomer | 33.29 | 283.0592 | C_16_H_12_O_5_ | 240.0405 | ＋ | - | - | Zong |
| C180 | Physcion isomer | 35.76 | 283.0584 | C_16_H_12_O_5_ | 240.0405 | ＋ | ＋ | ＋ | Fr3 |
| C181 | Alizarin | 35.97 | 239.0341 | C_14_H_8_O_4_ | 211.0382, 183.0453 | ＋ | ＋ | ＋ | Zong |
| C182 | Rhein | 36.14 | 283.0234 | C_15_H_8_O_6_ | 239.0333, 211.0378, 183.0444 | ＋ | ＋ | ＋ | Zong |
| C183 | Emodin | 37.25 | 269.0456 | C_15_H_10_O_5_ | 225.0547 | ＋ | ＋ | ＋ | Zong |

Note: the red text represented the compounds that can only be identified by offline 2D LPLC/HPLC coupled with MS technology; Regardless of whether MS is combined with offline 2D LC separation, other compounds can be identified; Zong represented the rhubarb extracting solution without offline 2D LC separation.

**Supplementary Table 2** The identification results of the prototypes and metabolites in RR, SR and CR.

| Num. | Name | RT (min) | [M-H]^-^ | PPM | Formula | MS/MS | RR  -P | RR  -U | SR  -P | SR  -U | CR  -P | CR  -U |
| --- | --- | --- | --- | --- | --- | --- | --- | --- | --- | --- | --- | --- |
| C1 | Gallic acid | 7.04 | 169.0138 | 3.85 | C_7_H_6_O_5_ | 125.0247 | ＋ | ＋ | ＋ | ＋ | ＋ | ＋ |
| C2 | Methylate-Gallic acid-*O*-GluA | 8.47 | 359.0577 | -8.04 | C_14_H_16_O_11_ | 183.0278, 124.0162 | ＋ | - | ＋ | + | ＋ | - |
| C3 | Methylate-Gallic acid | 9.01 | 183.0300 | 6.56 | C_8_H_8_O_5_ | 168.0066, 139.0390, 124.0145 | ＋ | - | - | - | - | - |
| C4 | Methylate-Gallic acid | 9.99 | 183.0295 | 3.83 | C_8_H_8_O_5_ | 168.0056, 139.0395, 124.0161 | ＋ | ＋ | ＋ | - | ＋ | - |
| C5 | Methylate-Gallic acid | 10.26 | 183.0294 | 3.28 | C_8_H_8_O_5_ | 168.0060, 139.0412, 124.0162 | ＋ | ＋ | ＋ | ＋ | ＋ | ＋ |
| C6 | Methylate-Gallic acid | 11.96 | 183.0295 | 3.83 | C_8_H_8_O_5_ | 168.0059, 139.0424, 124.0157 | ＋ | - | ＋ | - | ＋ | - |
| C7 | Methylate-Gallic acid | 12.23 | 183.0290 | 1.09 | C_8_H_8_O_5_ | 168.0060, 139.0415, 124.0163 | ＋ | - | ＋ | - | ＋ | - |
| C8 | Methylate-Gallic acid | 12.89 | 183.0299 | 6.01 | C_8_H_8_O_5_ | 168.0053, 124.0162 | - | - | - | ＋ | - | ＋ |
| C9 | Methylate-Gallic acid | 14.44 | 183.0300 | 6.56 | C_8_H_8_O_5_ | 168.0052, 124.0168 | - | + | ＋ | ＋ | ＋ | ＋ |
| C10 | Methylate-Gallic acid-*O*-SO_3_ | 10.33 | 262.9847 | -3.48 | C_8_H_8_SO_8_ | 183.0286, 168.0058, 124.0162 | ＋ | ＋ | ＋ | ＋ | ＋ | ＋ |
| C11 | Methylate-Gallic acid-*O*-SO_3_ | 14.41 | 262.9866 | 3.75 | C_8_H_8_SO_8_ | 183.0303, 168.0050, 124.0163 | - | ＋ | ＋ | ＋ | ＋ | ＋ |
| C12 | Dimethylate-Gallic acid-*O*-SO_3_ | 13.85 | 276.9997 | -5.65 | C_9_H_10_SO_8_ | 197.0440, 183.0261, 168.0012, 124.0122 | ＋ | ＋ | ＋ | ＋ | ＋ | - |
| C13 | Decarboxylate-Gallic acid | 7.04 | 125.0244 | 8.63 | C_6_H_6_O_3_ | - | ＋ | ＋ | ＋ | ＋ | ＋ | ＋ |
| C14 | Decarboxylate-Gallic acid | 7.88 | 125.0243 | 7.83 | C_6_H_6_O_3_ | - | ＋ | ＋ | ＋ | ＋ | ＋ | ＋ |
| C15 | Decarboxylate-Gallic acid | 8.83 | 125.0240 | 5.44 | C_6_H_6_O_3_ | - | - | ＋ | ＋ | ＋ | ＋ | ＋ |
| C16 | Decarboxylate-Gallic acid | 9.36 | 125.0243 | 7.83 | C_6_H_6_O_3_ | - | - | ＋ | - | ＋ | - | ＋ |
| C17 | Decarboxylate-Gallic acid | 11.27 | 125.0242 | 7.03 | C_6_H_6_O_3_ | - | - | - | - | ＋ | - | ＋ |
| C18 | Decarboxylate-Gallic acid-*O*-GluA | 6.83 | 301.0542 | -4.01 | C_12_H_14_O_9_ | 175.0244, 125.0248 | ＋ | ＋ | ＋ | ＋ | ＋ | ＋ |
| C19 | Gallic acid-*O*-GluA | 8.66 | 345.0427 | -7.35 | C_13_H_14_O_11_ | 169.0132, 125.0248 | ＋ | ＋ | ＋ | ＋ | ＋ | - |
| C20 | (Epi)catechin | 10.45 | 289.0694 | -4.38 | C_15_H_14_O_6_ | 271.0555, 245.0776 | ＋ | ＋ | ＋ | ＋ | ＋ | - |
| C21 | (Epi)catechin | 12.30 | 289.0722 | 5.31 | C_15_H_14_O_6_ | 271.0675, 245.0840 | ＋ | - | - | ＋ | - | + |
| C22 | Methylate-(Epi)catechin | 11.62 | 303.0871 | 2.59 | C_16_H_16_O_6_ | 285.0779, 259.0943 | ＋ | ＋ | - | - | - | - |
| C23 | Methylate-(Epi)catechin | 12.98 | 303.0844 | -6.32 | C_16_H_16_O_6_ | 285.0726, 259.0943 | ＋ | ＋ | ＋ | ＋ | ＋ | - |
| C24 | Methylate-(Epi)catechin | 14.29 | 303.0870 | 2.26 | C_16_H_16_O_6_ | 285.0708, 259.0948 | ＋ | ＋ | - | ＋ | - | - |
| C25 | Methylate-(Epi)catechin | 15.61 | 303.0875 | 3.91 | C_16_H_16_O_6_ | 285.0732, 259.0964 | - | ＋ | - | ＋ | - | ＋ |
| C26 | Methylate-(Epi)catechin-*O*-SO_3_ | 14.08 | 383.0393 | -10.00 | C_16_H_16_SO_9_ | 303.0837, 259.0929, 216.9814 | ＋ | ＋ | ＋ | ＋ | ＋ | ＋ |
| C27 | Methylate-(Epi)catechin-*O*-SO_3_ | 15.49 | 383.0388 | -11.30 | C_16_H_16_SO_9_ | 303.0820, 216.9839 | ＋ | ＋ | ＋ | ＋ | ＋ | ＋ |
| C28 | Methylate-(Epi)catechin-*O*-GluA | 11.63 | 479.1125 | -9.19 | C_22_H_24_O_12_ | 303.0847, 259.0936 | ＋ | ＋ | ＋ | ＋ | ＋ | - |
| C29 | Methylate-(Epi)catechin-*O*-GluA | 12.98 | 479.1146 | -7.94 | C_22_H_24_O_12_ | 303.0849, 259.0937 | ＋ | ＋ | ＋ | ＋ | ＋ | - |
| C30 | Methylate-(Epi)catechin-*O*-GluA | 14.32 | 479.1202 | 3.75 | C_22_H_24_O_12_ | 303.0878, 259.0981 | - | ＋ | - | ＋ | - | - |
| C31 | (Epi)catechin-*O*-GluA | 8.80 | 465.0984 | -9.36 | C_21_H_22_O_12_ | 289.0691, 245.0806 | ＋ | ＋ | ＋ | ＋ | ＋ | - |
| C32 | (Epi)catechin-*O*-GluA | 10.38 | 465.0999 | -6.13 | C_21_H_22_O_12_ | 289.0737, 245.0784 | ＋ | ＋ | ＋ | ＋ | ＋ | ＋ |
| C33 | (Epi)catechin-*O*-GluA | 12.49 | 465.0997 | -6.56 | C_21_H_22_O_12_ | 289.0686, 245.0779 | ＋ | ＋ | ＋ | ＋ | ＋ | - |
| C34 | (Epi)catechin-*O*-SO_3_ | 11.80 | 369.0255 | -5.36 | C_15_H_14_SO_9_ | 289.0678, 245.0872 | ＋ | ＋ | - | ＋ | - | ＋ |
| C35 | (Epi)catechin-*O*-SO_3_ | 22.56 | 369.0261 | -3.74 | C_15_H_14_SO_9_ | 289.0706, 245.0805 | - | - | ＋ | ＋ | - | - |
| C36 | Rhein | 36.00 | 283.0234 | -1.11 | C_15_H_8_O_6_ | 239.0307, 211.0374, 183.0438 | ＋ | ＋ | ＋ | ＋ | ＋ | ＋ |
| C37 | Rhein isomer | 21.61 | 283.0212 | -8.88 | C_15_H_8_O_6_ | 239.0309, 211.0368, 183.0452 | ＋ | ＋ | ＋ | - | ＋ | - |
| C38 | Rhein isomer | 23.63 | 283.0210 | -9.59 | C_15_H_8_O_6_ | 239.0324, 211.0376, 183.0449 | ＋ | ＋ | ＋ | ＋ | ＋ | ＋ |
| C39 | Hydroxyrhein | 25.49 | 299.0145 | -5.78 | C_15_H_8_O_7_ | 255.0260, 227.0310, 183.044 | ＋ | ＋ | ＋ | ＋ | ＋ | ＋ |
| C40 | Hydroxyrhein | 29.93 | 299.0191 | 1.58 | C_15_H_8_O_7_ | 255.0280, 227.0300, 211.0381 | ＋ | ＋ | ＋ | ＋ | ＋ | ＋ |
| C41 | Hydroxyrhein | 35.99 | 299.0194 | 2.58 | C_15_H_8_O_7_ | 255.0288, 227.0343, 211.0390 | ＋ | ＋ | ＋ | ＋ | ＋ | ＋ |
| C42 | Hydroxyrhein | 37.05 | 299.0180 | -2.10 | C_15_H_8_O_7_ | 255.0288, 227.0343, 211.0390 | - | ＋ | ＋ | ＋ | ＋ | ＋ |
| C43 | Rhein*-O*-SO_3_ | 23.50 | 362.9771 | -9.45 | C_15_H_8_SO_9_ | 283.0204, 239.0334, 211.0343, 183.0414 | ＋ | ＋ | ＋ | ＋ | ＋ | ＋ |
| C44 | Rhein*-O*-GluA | 21.56 | 459.0557 | -0.22 | C_21_H_16_O_12_ | 283.0199, 239.0293, 211.0442, 183.0435 | ＋ | ＋ | ＋ | ＋ | ＋ | ＋ |
| C45 | Hydroxyrhein-*O*-SO_3_ | 25.49 | 378.9759 | 1.21 | C_15_H_8_SO_10_ | 299.0140, 255.0261 | ＋ | ＋ | ＋ | ＋ | ＋ | ＋ |
| C46 | Hydroxyrhein-*O*-SO_3_ | 29.93 | 378.9748 | -1.70 | C_15_H_8_SO_10_ | 299.0153, 255.0271 | ＋ | ＋ | ＋ | ＋ | ＋ | ＋ |
| C47 | Methylate-Hydroxyrhein | 18.33 | 313.0345 | 0.71 | C_16_H_10_O_7_ | 233.0796, 189.0543 | ＋ | ＋ | ＋ | ＋ | ＋ | ＋ |
| C48 | Emodin | 37.25 | 269.0450 | 2.05 | C_15_H_10_O_5_ | 241.0466, 225.0526, 197.0591, 182.0368 | ＋ | ＋ | ＋ | ＋ | ＋ | ＋ |
| C49 | Emodin isomer | 18.34 | 269.0441 | -1.30 | C_15_H_10_O_5_ | 241.0489, 225.0533, 197.0559, 182.0387 | ＋ | - | ＋ | - | ＋ | - |
| C50 | Emodin isomer | 25.38 | 269.0431 | -5.02 | C_15_H_10_O_5_ | 241.0448, 225.0519, 197.0573, 182.0310 | ＋ | ＋ | ＋ | ＋ | ＋ | ＋ |
| C51 | Emodin isomer | 25.64 | 269.0462 | 6.51 | C_15_H_10_O_5_ | 241.0491, 225.0558, 197.0592, 182.0353 | - | ＋ | - | ＋ | + | ＋ |
| C52 | Emodin isomer | 26.31 | 269.0445 | 0.19 | C_15_H_10_O_5_ | 241.0475, 225.0525, 197.0622 | - | ＋ | - | ＋ | - | ＋ |
| C53 | Emodin isomer | 27.93 | 269.0443 | -0.56 | C_15_H_10_O_5_ | 241.0477, 225.0527, 197.0579, 182.0371 | ＋ | ＋ | ＋ | - | ＋ | - |
| C54 | Emodin isomer | 31.41 | 269.0452 | 2.79 | C_15_H_10_O_5_ | 241.0456, 225.0536, 197.0588, 182.0353 | ＋ | + | ＋ | - | ＋ | - |
| C55 | Carboxyemodin | 35.39 | 313.0348 | 1.66 | C_16_H_10_O_7_ | 269.0412, 225.0525 | ＋ | ＋ | ＋ | ＋ | ＋ | ＋ |
| C56 | Hydroxyemodin | 18.68 | 285.0399 | 1.88 | C_15_H_10_O_6_ | 241.0522, 211.0334 | ＋ | - | ＋ | - | ＋ | - |
| C57 | Hydroxyemodin | 22.54 | 285.0402 | 2.93 | C_15_H_10_O_6_ | 241.0413, 211.0375 | ＋ | ＋ | ＋ | ＋ | ＋ | ＋ |
| C58 | Hydroxyemodin | 25.73 | 285.0400 | 2.23 | C_15_H_10_O_6_ | 241.0463, 211.0369 | ＋ | - | ＋ | - | ＋ | + |
| C59 | Hydroxyemodin | 26.09 | 285.0397 | 1.18 | C_15_H_10_O_6_ | 241.0504, 211.0386 | - | ＋ | ＋ | ＋ | + | ＋ |
| C60 | Hydroxyemodin | 31.77 | 285.0406 | 4.34 | C_15_H_10_O_6_ | 241.0513, 211.0370 | ＋ | ＋ | ＋ | ＋ | ＋ | ＋ |
| C61 | Hydroxyemodin | 33.54 | 285.0401 | 2.58 | C_15_H_10_O_6_ | 241.0395, 211.0382 | ＋ | - | - | - | - | - |
| C62 | Hydroxyemodin | 35.29 | 285.0402 | 2.93 | C_15_H_10_O_6_ | 241.0414, 211.0373 | ＋ | ＋ | ＋ | ＋ | ＋ | ＋ |
| C63 | Hydroxyemodin*-O*-GluA | 22.60 | 461.0715 | 0.10 | C_21_H_18_O_12_ | 285.0351, 257.0409, 241.0453, 211.0353 | ＋ | ＋ | ＋ | ＋ | ＋ | ＋ |
| C64 | Hydroxyemodin*-O*-SO_3_ | 25.34 | 364.9969 | 1.98 | C_15_H_10_SO_9_ | 285.0360, 241.0469, 211.0371 | ＋ | - | ＋ | - | ＋ | - |
| C65 | Hydroxyemodin*-O*-SO_3_ | 26.02 | 364.9967 | 1.43 | C_15_H_10_SO_9_ | 285.0342, 241.0431, 211.0405 | ＋ | + | ＋ | + | ＋ | + |
| C66 | Emodin-*O*-GluA | 19.33 | 445.0744 | -4.80 | C_21_H_18_O_11_ | 269.0429, 241.0499 | ＋ | ＋ | ＋ | - | ＋ | - |
| C67 | Emodin-*O*-SO_3_ | 20.24 | 348.9989 | -6.78 | C_21_H_18_O_11_ | 269.0416, 241.0465, 225.0527 | ＋ | ＋ | ＋ | ＋ | ＋ | ＋ |
| C68 | Emodin-*O*-SO_3_ | 21.98 | 348.9979 | -9.64 | C_15_H_10_SO_8_ | 269.0416, 241.0465, 225.0498 | ＋ | ＋ | ＋ | ＋ | ＋ | ＋ |
| C69 | Emodin-*O*-SO_3_ | 30.06 | 348.9987 | -7.35 | C_15_H_10_SO_8_ | 269.0411, 241.0471, 225.0512 | ＋ | - | ＋ | + | ＋ | + |
| C70 | Emodin-*O*-SO_3_ | 32.05 | 348.9980 | -9.35 | C_15_H_10_SO_8_ | 269.0418, 241.0445, 225.0539 | ＋ | + | ＋ | + | ＋ | + |
| C71 | Emodin-*O*-SO_3_ | 36.08 | 348.9975 | -13.93 | C_15_H_10_SO_8_ | 269.0435, 241.0494, 225.0566 | + | - | - | - | - | - |
| C72 | Emodin-*O*-GluA-SO_3_ | 16.01 | 525.0341 | 1.42 | C_21_H_18_SO_14_ | 445.0796, 349.0013, 269.0406 | ＋ | ＋ | ＋ | - | ＋ | - |
| C73 | Emodin-*O*-GluA-SO_3_ | 18.33 | 525.0301 | -6.19 | C_21_H_18_SO_14_ | 445.0677, 348.9986, 269.0414, 225.0568 | ＋ | ＋ | ＋ | ＋ | ＋ | ＋ |
| C74 | Aloe-emodin | 31.96 | 269.0432 | -4.65 | C_15_H_10_O_5_ | 239.0321 | ＋ | - | ＋ | - | ＋ | - |
| C75 | Aloe-emodin isomer | 19.38 | 269.0424 | -7.62 | C_15_H_10_O_5_ | 239.0306 | ＋ | ＋ | ＋ | - | ＋ | - |
| C76 | Aloe-emodin isomer | 20.43 | 269.0431 | -5.02 | C_15_H_10_O_5_ | 239.0305 | ＋ | ＋ | ＋ | ＋ | ＋ | ＋ |
| C77 | Aloe-emodin isomer | 21.98 | 269.0429 | -5.76 | C_15_H_10_O_5_ | 239.0301 | ＋ | ＋ | ＋ | ＋ | ＋ | ＋ |
| C78 | Physcion | 30.52 | 283.0605 | 1.41 | C_16_H_12_O_5_ | 240.0455 | ＋ | ＋ | ＋ | - | ＋ | - |
| C79 | Physcion isomer | 31.59 | 283.0584 | -6.01 | C_16_H_12_O_5_ | 240.0375 | ＋ | ＋ | ＋ | - | ＋ | - |
| C80 | Physcion isomer | 32.86 | 283.0594 | -2.47 | C_16_H_12_O_5_ | 240.0416 | ＋ | ＋ | - | ＋ | - | ＋ |
| C81 | Physcion isomer | 36.42 | 283.0607 | 2.12 | C_16_H_12_O_5_ | 240.0400 | ＋ | ＋ | ＋ | ＋ | ＋ | ＋ |
| C82 | Hydroxyphyscion | 23.63 | 299.0546 | -1.39 | C_16_H_12_O_6_ | 256.0371 | ＋ | ＋ | ＋ | ＋ | - | ＋ |
| C83 | Hydroxyphyscion | 25.87 | 299.0539 | -3.73 | C_16_H_12_O_6_ | 256.0370 | - | ＋ | ＋ | ＋ | ＋ | ＋ |
| C84 | Hydroxyphyscion | 27.91 | 299.0547 | -1.05 | C_16_H_12_O_6_ | 256.0359 | - | ＋ | ＋ | ＋ | ＋ | - |
| C85 | Hydroxyphyscion | 31.50 | 299.0544 | -2.06 | C_16_H_12_O_6_ | 256.0370 | - | ＋ | - | - | - | - |
| C86 | Hydroxyphyscion | 34.37 | 299.0560 | 3.30 | C_16_H_12_O_6_ | 256.0357 | ＋ | - | ＋ | - | ＋ | - |
| C87 | Hydroxyphyscion | 34.98 | 299.0542 | -2.72 | C_16_H_12_O_6_ | 256.0355 | ＋ | ＋ | - | - | - | - |
| C88 | Hydroxyphyscion | 37.05 | 299.0554 | 1.29 | C_16_H_12_O_6_ | 256.0355 | - | ＋ | - | ＋ | - | ＋ |
| C89 | Hydroxyphyscion | 37.45 | 299.0533 | -5.73 | C_16_H_12_O_6_ | 256.0366 | - | ＋ | - | ＋ | - | ＋ |
| C90 | Physcion-*O*-GluA | 30.49 | 459.0901 | -4.55 | C_22_H_20_O_11_ | 283.0561, 240.0380 | ＋ | ＋ | ＋ | - | ＋ | - |
| C91 | Physcion-*O*-GluA | 31.63 | 459.0900 | -4.77 | C_22_H_20_O_11_ | 283.0552, 240.0364 | ＋ | ＋ | ＋ | - | ＋ | - |
| C92 | Physcion-*O*-SO_3_ | 33.77 | 363.0153 | -4.45 | C_16_H_12_SO_8_ | 283.0597, 240.0419 | - | ＋ | - | ＋ | - | ＋ |
| C93 | Physcion-*O*-SO_3_ | 21.67 | 363.0160 | -2.52 | C_16_H_12_SO_8_ | 283.0585, 240.0392 | - | ＋ | - | ＋ | - | ＋ |
| C94 | Physcion-*O*-SO_3_ | 20.88 | 363.0163 | -1.69 | C_16_H_12_SO_8_ | 283.0591, 240.0458 | ＋ | ＋ | - | ＋ | - | ＋ |
| C95 | Physcion-*O*-SO_3_ | 22.90 | 363.0159 | -2.79 | C_16_H_12_SO_8_ | 283.0586, 240.0432 | - | ＋ | - | ＋ | - | ＋ |
| C96 | Hydroxyphyscion-*O*-SO_3_ | 34.37 | 379.0098 | -5.35 | C_16_H_12_SO_9_ | 299.0515 | ＋ | - | ＋ | - | ＋ | - |
| C97 | Hydroxyphyscion-*O*-SO_3_ | 35.12 | 379.0102 | -4.30 | C_16_H_12_SO_9_ | 299.0529 | - | ＋ | - | ＋ | - | ＋ |
| C98 | Hydroxyphyscion-*O*-SO_3_ | 28.53 | 379.0097 | -5.62 | C_16_H_12_SO_9_ | 299.0528 | - | ＋ | - | ＋ | - | ＋ |
| C99 | Hydroxyphyscion-*O*-SO_3_ | 25.83 | 379.0098 | -5.35 | C_16_H_12_SO_9_ | 299.0539 | ＋ | ＋ | - | ＋ | ＋ | ＋ |
| C100 | Hydroxyphyscion-*O*-SO_3_ | 23.28 | 379.0103 | -4.03 | C_16_H_12_SO_9_ | 299.0529 | ＋ | ＋ | ＋ | ＋ | ＋ | ＋ |
| C101 | Hydroxyphyscion-*O*-SO_3_ | 23.57 | 379.0102 | -4.30 | C_16_H_12_SO_9_ | 299.0536 | - | ＋ | - | ＋ | - | ＋ |
| C102 | 6-Methylrhein | 32.63 | 297.0379 | -4.93 | C_16_H_10_O_6_ | 253.0491, 225.0528 | ＋ | ＋ | ＋ | ＋ | ＋ | ＋ |
| C103 | 6-Methylrhein isomer | 35.98 | 297.0381 | -4.26 | C_16_H_10_O_6_ | 253.0452, 225.0511 | ＋ | ＋ | ＋ | ＋ | ＋ | ＋ |
| C104 | Chrysophanol | 28.16 | 253.0494 | -0.54 | C_15_H_10_O_4_ | 225.053 | ＋ | ＋ | ＋ | ＋ | ＋ | ＋ |
| C105 | Chrysophanol isomer | 20.36 | 253.0497 | 0.65 | C_15_H_10_O_4_ | 225.0560 | - | ＋ | - | ＋ | - | ＋ |
| C106 | Chrysophanol isomer | 21.09 | 253.0473 | -8.83 | C_15_H_10_O_4_ | 225.0526 | ＋ | ＋ | ＋ | ＋ | - | ＋ |
| C107 | Chrysophanol isomer | 23.99 | 253.0503 | 3.02 | C_15_H_10_O_4_ | 225.0549 | - | ＋ | - | ＋ | - | ＋ |
| C108 | Chrysophanol isomer | 25.31 | 253.0473 | -8.83 | C_15_H_10_O_4_ | 225.0524 | ＋ | ＋ | ＋ | ＋ | ＋ | ＋ |
| C109 | Chrysophanol isomer | 27.10 | 253.0471 | -9.62 | C_15_H_10_O_4_ | 225.0525 | ＋ | ＋ | ＋ | - | ＋ | - |
| C110 | Chrysophanol isomer | 27.43 | 253.0477 | -7.25 | C_15_H_10_O_4_ | 225.0528 | ＋ | ＋ | ＋ | - | ＋ | - |
| C111 | Chrysophanol isomer | 32.63 | 253.0491 | -1.72 | C_15_H_10_O_4_ | 225.0520 | ＋ | ＋ | ＋ | ＋ | ＋ | ＋ |
| C112 | Chrysophanol isomer | 35.98 | 253.0468 | -10.81 | C_15_H_10_O_4_ | 225.0505 | ＋ | - | ＋ | - | ＋ | - |
| C113 | Chrysophanol isomer | 36.95 | 253.0498 | 1.05 | C_15_H_10_O_4_ | 225.0534 | - | ＋ | - | ＋ | - | ＋ |
| C114 | Chrysophanol isomer | 38.18 | 253.0497 | 0.65 | C_15_H_10_O_4_ | 225.0538 | - | ＋ | - | ＋ | - | ＋ |
| C115 | Chrysophanol-*O*-GluA | 27.17 | 429.0795 | -4.95 | C_21_H_18_O_10_ | 253.0472, 225.0528 | ＋ | ＋ | ＋ | - | ＋ | - |
| C116 | Chrysophanol-*O*-GluA | 27.51 | 429.0786 | -7.05 | C_21_H_18_O_10_ | 253.0454, 225.0545 | ＋ | ＋ | ＋ | - | ＋ | - |
| C117 | Chrysophanol-*O*-SO_3_ | 20.11 | 333.0056 | -2.25 | C_15_H_10_SO_7_ | 253.0489, 225.0581 | ＋ | ＋ | ＋ | ＋ | ＋ | ＋ |
| C118 | Chrysophanol-*O*-SO_3_ | 21.00 | 333.0087 | 7.06 | C_15_H_10_SO_7_ | 253.0501, 225.0544 | - | ＋ | - | ＋ | - | ＋ |
| C119 | Chrysophanol-*O*-SO_3_ | 28.16 | 333.0070 | 1.95 | C_15_H_10_SO_7_ | 253.0475, 225.0537 | ＋ | ＋ | ＋ | ＋ | ＋ | ＋ |
| C120 | Methylate-Chrysophanol | 31.67 | 267.0640 | -4.44 | C_16_H_12_O_4_ | 225.0515 | ＋ | ＋ | ＋ | ＋ | ＋ | ＋ |
| C121 | Methylate-Chrysophanol | 36.18 | 267.0645 | -2.57 | C_16_H_12_O_4_ | 239.0671 | ＋ | ＋ | ＋ | ＋ | ＋ | ＋ |
| C122 | Dihydrodeoxygenate- Chrysophanol-*O*-GluA | 20.02 | 417.1189 | 2.14 | C_21_H_22_O_9_ | 241.0839, 135.0453, 121.0294 | ＋ | ＋ | ＋ | ＋ | ＋ | ＋ |
| C123 | Chrysazin | 21.61 | 239.0316 | -9.56 | C_14_H_8_O_4_ | 211.0367, 183.0429 | ＋ | ＋ | ＋ | - | ＋ | ＋ |
| C124 | Chrysazin isomer or Alizarin isomer | 23.56 | 239.0319 | -8.31 | C_14_H_8_O_4_ | 211.0371, 183.0447 | ＋ | ＋ | ＋ | ＋ | ＋ | ＋ |
| C125 | Alizarin | 35.98 | 239.0326 | -5.38 | C_14_H_8_O_4_ | 211.0367, 183.0439 | ＋ | ＋ | ＋ | ＋ | ＋ | ＋ |
| C126 | Emodin Anthrone or its isomer | 20.49 | 255.0629 | -8.96 | C_15_H_12_O_4_ | 213.0542, 145.0655 | ＋ | - | ＋ | - | ＋ | - |
| C127 | Emodin Anthrone or its isomer | 21.00 | 255.0627 | -9.74 | C_15_H_12_O_4_ | 213.0536, 145.0674 | ＋ | ＋ | ＋ | ＋ | ＋ | ＋ |
| C128 | Emodin Anthrone or its isomer | 23.66 | 255.0657 | 2.02 | C_15_H_12_O_4_ | 213.0554, 145.0668 | - | ＋ | - | ＋ | - | ＋ |
| C129 | Emodin Anthrone or its isomer | 31.21 | 255.0651 | -0.34 | C_15_H_12_O_4_ | 213.0527, 145.0661 | - | ＋ | - | ＋ | - | ＋ |
| C130 | Torachrysone or its isomer | 24.92 | 245.0792 | -6.67 | C_14_H_14_O_4_ | 230.0562, 215.0337, 159.0440 | ＋ | ＋ | ＋ | - | ＋ | + |
| C131 | Torachrysone or its isomer | 27.03 | 245.0802 | -2.59 | C_14_H_14_O_4_ | 230.0558, 215.0329, 159.0441 | ＋ | ＋ | ＋ | ＋ | ＋ | ＋ |
| C132 | Torachrysone or its isomer | 35.58 | 245.0809 | 0.26 | C_14_H_14_O_4_ | 230.0558, 215.0328, 159.0434 | ＋ | ＋ | ＋ | ＋ | ＋ | ＋ |
| C133 | Torachrysone or its isomer | 36.66 | 245.0796 | -5.04 | C_14_H_14_O_4_ | 230.0569, 215.0325, 159.0439 | ＋ | ＋ | ＋ | ＋ | ＋ | ＋ |
| C134 | Carbonylate-Torachrysone | 22.95 | 259.0594 | -2.70 | C_14_H_12_O_5_ | 231.0643, 216.0398, 188.0473, 159.0428 | ＋ | ＋ | ＋ | ＋ | ＋ | ＋ |
| C135 | Carbonylate-Torachrysone | 27.64 | 259.0591 | -3.86 | C_14_H_12_O_5_ | 215.0681, 173.0585 | ＋ | ＋ | - | - | - | ＋ |
| C136 | Demethylate-Torachrysone-*O*-GluA | 13.76 | 407.0954 | -4.60 | C_19_H_20_O_10_ | 231.0640 | ＋ | ＋ | ＋ | - | ＋ | - |
| C137 | Demethylate-Torachrysone-*O*-GluA | 22.46 | 407.0947 | -6.32 | C_19_H_20_O_10_ | 231.0644 | ＋ | - | - | - | - | - |
| C138 | Hydroxytorachrysone-*O*-SO_3_ | 23.50 | 341.0338 | 3.62 | C_14_H_14_SO_8_ | 261.0721, 246.0487, 175.0370 | ＋ | - | ＋ | - | ＋ | - |
| C139 | Hydroxytorachrysone-*O*-SO_3_ | 25.31 | 341.0307 | -5.47 | C_14_H_14_SO_8_ | 261.0728, 246.0496, 231.0275, 175.0398 | ＋ | ＋ | ＋ | ＋ | ＋ | ＋ |
| C140 | Hydroxytorachrysone | 25.31 | 261.0737 | -7.85 | C_14_H_14_O_5_ | 246.0496, 231.0280, 175.0386 | ＋ | ＋ | ＋ | ＋ | ＋ | ＋ |
| C141 | Methylate-Hydroxytorachrysone | 17.44 | 275.0920 | 2.18 | C_15_H_16_O_5_ | 231.0671, 189.0565 | - | ＋ | - | ＋ | - | ＋ |
| C142 | Methylate-Hydroxytorachrysone | 19.33 | 275.0914 | 0.00 | C_15_H_16_O_5_ | 231.0646, 189.0551 | - | ＋ | - | ＋ | - | ＋ |
| C143 | Methylate-Hydroxytorachrysone | 20.67 | 275.0917 | 1.09 | C_15_H_16_O_5_ | 231.0683, 189.0558 | - | ＋ | - | ＋ | - | ＋ |
| C144 | Methylate-Hydroxytorachrysone | 24.58 | 275.0930 | 5.82 | C_15_H_16_O_5_ | 231.1005 | ＋ | ＋ | ＋ | ＋ | ＋ | - |
| C145 | Methylate-Hydroxytorachrysone | 30.75 | 275.0905 | -3.27 | C_15_H_16_O_5_ | 231.0999 | ＋ | - | ＋ | - | ＋ | - |
| C146 | Torachrysone-*O*-GluA | 24.92 | 421.1097 | -7.65 | C_20_H_22_O_10_ | 245.0788, 230.0553, 215.0334, 159.0449 | ＋ | ＋ | ＋ | - | ＋ | - |
| C147 | Torachrysone-*O*-SO_3_ | 27.03 | 325.0352 | -7.54 | C_14_H_14_SO_7_ | 245.0786, 230.0546, 215.0326, 159.0440 | ＋ | ＋ | ＋ | ＋ | ＋ | ＋ |
| C148 | Demethylate-Torachrysone | 16.58 | 231.0648 | -1.67 | C_13_H_12_O_4_ | 145.0603 | ＋ | ＋ | ＋ | ＋ | ＋ | ＋ |
| C149 | Demethylate-Torachrysone | 21.09 | 231.0633 | -8.16 | C_13_H_12_O_4_ | 145.0653 | ＋ | - | - | - | - | - |
| C150 | Demethylate-Torachrysone | 22.67 | 231.0635 | -7.29 | C_13_H_12_O_4_ | 145.0650 | ＋ | ＋ | ＋ | ＋ | ＋ | ＋ |
| C151 | Demethylate-Torachrysone | 24.04 | 231.0634 | -7.73 | C_13_H_12_O_4_ | 145.0629 | ＋ | ＋ | - | ＋ | - | ＋ |
| C152 | Caffeic acid | 15.23 | 179.0352 | 7.34 | C_9_H_8_O_4_ | 135.0453, 107.0500 | - | - | - | ＋ | - | ＋ |
| C153 | Caffeic acid isomer | 12.83 | 179.0352 | 7.34 | C_9_H_8_O_4_ | 135.0443, 107.0493 | - | ＋ | - | ＋ | - | ＋ |
| C154 | Caffeic acid isomer | 13.99 | 179.0342 | 1.76 | C_9_H_8_O_4_ | 135.0454, 107.0479 | ＋ | - | ＋ | - | ＋ | - |
| C155 | Caffeic acid isomer | 14.28 | 179.0349 | 5.67 | C_9_H_8_O_4_ | 135.0462, 107.0523 | - | ＋ | - | ＋ | - | ＋ |
| C156 | Caffeic acid isomer | 16.24 | 179.0348 | 5.11 | C_9_H_8_O_4_ | 135.0457, 107.0501 | - | ＋ | - | ＋ | - | ＋ |
| C157 | Caffeic acid isomer | 16.79 | 179.0350 | 6.23 | C_9_H_8_O_4_ | 135.0451, 107.0497 | - | ＋ | - | ＋ | - | ＋ |
| C158 | Methylate-Caffeic acid | 14.30 | 193.0497 | 0.85 | C_10_H_10_O_4_ | 149.0595 | ＋ | ＋ | ＋ | ＋ | ＋ | - |
| C159 | Methylate-Caffeic acid | 16.48 | 193.0502 | 3.44 | C_10_H_10_O_4_ | 149.0597 | - | ＋ | - | ＋ | - | ＋ |
| C160 | Methylate-Caffeic acid | 17.49 | 193.0505 | 5.00 | C_10_H_10_O_4_ | 149.0594 | - | ＋ | - | ＋ | - | ＋ |
| C161 | Methylate-Caffeic acid | 18.56 | 193.0502 | 3.44 | C_10_H_10_O_4_ | 149.0588 | - | ＋ | - | ＋ | - | ＋ |
| C162 | Methylate-Caffeic acid | 20.97 | 193.0502 | 3.44 | C_10_H_10_O_4_ | 149.0595 | - | ＋ | - | ＋ | - | ＋ |
| C163 | Methylate-Caffeic acid-*O*-SO_3_ | 14.30 | 273.0074 | 3.85 | C_10_H_10_SO_7_ | 193.0490, 149.0588 | ＋ | ＋ | ＋ | ＋ | ＋ | - |
| C164 | Methylate-Caffeic acid-*O*-SO_3_ | 15.80 | 273.0068 | 1.65 | C_10_H_10_SO_7_ | 193.0500, 149.0621 | - | ＋ | - | ＋ | - | ＋ |
| C165 | Methylate-Caffeic acid-*O*-SO_3_ | 16.48 | 273.0068 | 1.65 | C_10_H_10_SO_7_ | 193.0507, 149.0558 | - | ＋ | - | ＋ | - | ＋ |
| C166 | Methylate-Caffeic acid-*O*-SO_3_ | 17.49 | 273.0068 | 1.65 | C_10_H_10_SO_7_ | 193.0505, 149.0606 | - | ＋ | - | ＋ | - | ＋ |
| C167 | Caffeic acid-*O*-SO_3_ | 13.99 | 258.9891 | -6.18 | C_9_H_8_O_7_S | 179.0344, 135.0443, 107.0508 | ＋ | - | ＋ | - | ＋ | - |
| C168 | Dehydrogenate-Caffeic acid | 14.79 | 177.0185 | 1.50 | C_9_H_6_O_4_ | 133.0293, 105.0349 | ＋ | ＋ | ＋ | - | ＋ | - |
| C169 | Dehydrogenate-Caffeic acid | 19.70 | 177.0190 | 4.32 | C_9_H_6_O_4_ | 133.0291, 105.0343 | - | ＋ | - | ＋ | - | ＋ |
| C170 | Cinnamic acid | 17.30 | 147.0445 | 3.02 | C_9_H_8_O_2_ | 103.0563 | ＋ | ＋ | ＋ | ＋ | ＋ | - |
| C171 | Cinnamic acid isomer | 7.73 | 147.0453 | 8.46 | C_9_H_8_O_2_ | 103.0556 | ＋ | ＋ | ＋ | ＋ | ＋ | ＋ |
| C172 | Cinnamic acid isomer | 24.19 | 147.0435 | -3.78 | C_9_H_8_O_2_ | 103.0556 | ＋ | ＋ | ＋ | ＋ | ＋ | - |
| C173 | Hydroxycinnamic acid | 5.11 | 163.0395 | 3.25 | C_9_H_8_O_3_ | 119.0500 | ＋ | ＋ | ＋ | - | ＋ | - |
| C174 | Hydroxycinnamic acid | 13.99 | 163.0397 | 4.47 | C_9_H_8_O_3_ | 119.0499 | ＋ | ＋ | ＋ | ＋ | ＋ | ＋ |
| C175 | Hydroxycinnamic acid | 14.82 | 163.0398 | 5.09 | C_9_H_8_O_3_ | 119.0509 | - | - | - | - | - | ＋ |
| C176 | Hydroxycinnamic acid | 17.90 | 163.0396 | 3.86 | C_9_H_8_O_3_ | 119.0505 | ＋ | ＋ | ＋ | ＋ | ＋ | ＋ |
| C177 | Hydrate-Hydroxycinnamic acid | 11.72 | 181.0498 | 1.46 | C_9_H_10_O_4_ | 163.0399, 119.0503 | ＋ | ＋ | ＋ | - | ＋ | - |
| C178 | Hydroxycinnamic acid-*O*-SO_3_ | 13.08 | 242.9968 | 4.18 | C_9_H_8_SO_6_ | 163.0402, 119.0495 | - | - | - | ＋ | - | ＋ |
| C179 | Hydroxycinnamic acid-*O*-SO_3_ | 13.99 | 242.9969 | 4.59 | C_9_H_8_SO_6_ | 163.0393, 119.0502 | ＋ | - | ＋ | - | ＋ | ＋ |
| C180 | Malic acid | 3.53 | 133.0121 | -7.89 | C_4_H_6_O_5_ | 115.0012 | ＋ | - | ＋ | - | ＋ | - |
| C181 | Resveratrol 4'-*O*-*β*-D-glucopyranoside | 24.70 | 389.1239 | 2.07 | C_20_H_22_O_8_ | 227.0680 | ＋ | - | - | - | - | - |
| C182 | Aloesol | 15.21 | 233.0803 | -2.30 | C_13_H_14_O_4_ | 189.0565, 149.0237, 105.0352 | ＋ | ＋ | ＋ | - | ＋ | - |
| C183 | Aloesol | 18.33 | 233.0798 | -4.44 | C_13_H_14_O_4_ | 189.0540, 149.0222, 105.0342 | ＋ | ＋ | ＋ | ＋ | ＋ | ＋ |
| C184 | Aloesol | 21.82 | 233.0794 | -6.16 | C_13_H_14_O_4_ | 189.0548, 149.0230, 105.0345 | ＋ | ＋ | ＋ | ＋ | ＋ | ＋ |
| C185 | Coumalic acid isomer | 5.11 | 163.0395 | 3.25 | C_9_H_8_O_3_ | 119.0500 | ＋ | ＋ | ＋ | - | ＋ | - |
| C186 | Coumalic acid isomer | 13.99 | 163.0397 | 4.47 | C_9_H_8_O_3_ | 119.0502 | ＋ | ＋ | ＋ | ＋ | ＋ | ＋ |
| C187 | Coumalic acid | 17.90 | 163.0396 | 3.86 | C_9_H_8_O_3_ | 119.0505 | ＋ | ＋ | ＋ | ＋ | ＋ | - |
| C188 | Methoxy-galloylglucose | 6.59 | 345.0817 | 0.22 | C_14_H_18_O_10_ | 183.0285, 168.0050 | - | ＋ | - | ＋ | - | - |
| C189 | Methoxy-galloylglucose | 7.51 | 345.0830 | 3.99 | C_14_H_18_O_10_ | 183.0277, 168.0037 | - | ＋ | - | ＋ | - | - |
| C190 | Methoxy-galloylglucose | 8.02 | 345.0824 | 2.25 | C_14_H_18_O_10_ | 183.0308, 168.0080 | - | ＋ | - | ＋ | - | - |
| C191 | Methoxy-galloylglucose | 9.01 | 345.0824 | 2.25 | C_14_H_18_O_10_ | 183.0287, 168.0052 | ＋ | - | - | - | - | - |
| C192 | Methoxy-galloylglucose | 11.19 | 345.0801 | -4.41 | C_14_H_18_O_10_ | 183.0313, 168.0059 | - | ＋ | - | ＋ | - | ＋ |
| C193 | Galloylglucose | 3.76 | 331.0644 | -4.75 | C_13_H_16_O_10_ | 211.0213, 169.0128, 151.0037, 125.0237 | ＋ | - | ＋ | ＋ | ＋ | - |
| C194 | Galloylglucose | 5.11 | 331.0649 | -3.24 | C_13_H_16_O_10_ | 211.0211, 169.0146, 151.0037, 125.0246 | ＋ | - | - | - | ＋ | - |
| C195 | Galloylglucose | 5.38 | 331.0649 | -3.24 | C_13_H_16_O_10_ | 211.0287, 169.0146, 151.0023, 125.0213 | ＋ | ＋ | - | - | ＋ | - |
| C196 | Galloylglucose | 6.23 | 331.0653 | -2.03 | C_13_H_16_O_10_ | 211.0231, 169.0140, 151.0004, 125.0247 | ＋ | ＋ | ＋ | ＋ | ＋ | ＋ |

Note: P: plasma; U: urine.
